# Tolerance to total dissolved gas supersaturation in Atlantic salmon, brown trout, rainbow trout, and European minnow

**DOI:** 10.1371/journal.pone.0342556

**Published:** 2026-02-11

**Authors:** Trond Einar Isaksen, Ulrich Pulg, Robert J. Lennox, Sebastian F. Stranzl, Einar Bye-Ingebrigtsen, Reidar Handegård, Gaute Velle

**Affiliations:** 1 Department Ocean & Environment, NORCE Norwegian Research Centre, Bergen, Norway; 2 Stiftelsen Industrilaboratoriet (ILAB), Bergen, Norway; Universität für Bodenkultur Wien: Universitat fur Bodenkultur Wien, AUSTRIA

## Abstract

Gas bubble disease is a major concern for aquatic animals in rivers downstream of hydroelectric power plants, where water can become supersaturated with dissolved gas. While the disease is well documented in Pacific salmonids (*Oncorhynchus* spp.), its effects on juvenile Atlantic salmonids (*Salmo* spp.) and non-salmonid fish in Europe are limited or unknown. To assess the impacts of gas bubble disease in Norwegian rivers, experimental trials exposed juvenile Atlantic salmonids (*Salmo salar* and *Salmo trutta*) and adult European minnows (*Phoxinus phoxinus*) to dissolved gas supersaturation ranging from 100% (normal) to 120%. A total of 1 440 fish were included in the trials, comprising Atlantic salmon larvae (alevin, *n* = 250; fry, *n* = 250), salmon parr (*S. salar*, *n* = 325), brown trout parr (*S. trutta*, *n* = 340), and minnow (*n* = 250). The study also included a repeated trial with salmon parr (*n* = 25) and a reference trial with rainbow trout parr (*Oncorhynchus mykiss*, *n* = 25). Results showed both intra- and interspecific differences in tolerance to total dissolved gas supersaturation. Atlantic salmon larvae (alevins and fry) and European minnows tolerated higher supersaturation, showing low mortality and only subacute gas bubble disease pathogenesis at levels up to 120% total dissolved gas supersaturation. Acute disease occurred only in larger salmonid parr at 115–120%, with gas emboli in gills and subcutaneous emphysema appearing within 1–6 hours. At 120%, mortality rates were significantly higher (p < 0.003) in salmon and brown trout parr compared to rainbow trout parr, ranging from 1.2–7.5 dead or moribund fish per hour (LT_50_ in 4–15 hours) versus 0.5 per hour in rainbow trout (LT_50_ in 25 hours). Lethal time for 50% mortality (LT_50_) was used as a criterion for terminating the trials. Recovery tests were conducted only on salmon larvae and brown trout in our study, and the results indicated partial recovery following exposure to sublethal gas supersaturation. These findings define lethal and sublethal thresholds of gas supersaturation for multiple species and life stages, highlight lower mortality rate in rainbow trout compared to Atlantic salmonids, and present the first documented effects on the non-salmonid European minnow (Cypriniformes). The results provide a basis for assessing potential ecological impacts of total dissolved gas supersaturation in Norwegian and European rivers.

## Introduction

Gas bubble disease (GBD; syn. gas bubble trauma) is a non-infectious disease harmful to aquatic animals. The pathogenesis of the disease among fish is well known and is typically characterized by the formation of gas bubbles in the tissue (subcutaneous emphysema) and in the blood vessels (gas embolism) causing blockage of blood flow, exophthalmia, and haemorrhages [1–8]. Severe GBD affects swimming performance and the predator avoidance response in juvenile salmonids, making fish more susceptible to predation [9–11].

GBD is caused by total dissolved gas supersaturation (TDGS) in water [1]. At sea level (water surface), TDGS is defined as the difference between the total gas pressure (TGP) in the water and the barometric pressure, occurring when TGP exceeds barometric pressure [2]. TDGS can result from a variety of physical and biological processes, and the mechanisms responsible for the formation of gas supersaturation are described in detail by Colt [2], based on Henry’s law. TDGS occurs when the water’s capacity to dissolve gas is reduced, i.e., when water is heated, or pressure decreases. TDGS can arise naturally in rivers when cold water becomes rapidly heated, if air is entrained in deep water below rapids, or because of high photosynthetic activity. TDGS in river systems can also result from anthropogenic activities, and high levels caused by hydroelectric power plants or dams pose a significant threat to fish populations [5,8,12–20] and benthic invertebrate populations [21].

Hydropower constitutes more than 90% of the total electrical power production in Norway. More than 1700 hydropower plants generate an annual production of more than 136 TWh (Norwegian Water authorities; www.nve.no, 8/1/2024). Surveys have registered DGS and signs of acute gas bubble disease and mortality among Atlantic salmon (*Salmo salar*) and brown trout (*Salmo trutta*) in several Norwegian rivers regulated for hydropower [22–24]. Pulg et al. [19] documented TDGS being caused by at least 17 hydropower stations in Norway and identified more than 800 being at risk of generating episodes of TDGS. Acute fish mortality due to GBD was observed in 8 of the 17 stations, specifically affecting Atlantic salmon and brown trout. The study also found that TDGS exceeded 200%, with regular occurrences of TDGS surpassing 110% for several weeks each year. In Norway, 28% of hydropower plants pose a high risk of producing TDGS, while 80% of plants in Austria and 66% of plants in Germany pose a moderate risk of producing TDGS [19]. Still, there is no systematic environmental monitoring of hydropower induced gas supersaturation in freshwaters in Norway or elsewhere in Europe despite exceptional research initiatives.

Experimental studies of juvenile salmonids (parr) have shown that GBD can occur at experienced total TDGS of 102% with significant increased severity and mortality for TDG levels >110% [8,15,25–27]. Most of these studies concern Pacific salmonids (*Oncorhynchus* spp.), common in regulated rivers and lakes in North America.

Based on these studies, threshold values ranging from 103% to 110% for lethal levels of TDGS have been established, followed by guidelines for TDGS in rivers in Canada (the Canadian Council of Ministers of the Environment) and USA (US Environmental Protection Agency), respectively. The guidelines are established to avoid lethal TDGS. However, sublethal levels of TDGS can also have serious consequences for fish including abnormal swimming ability, reduced growth, and an immune response [3,28,29]. Long-term exposure to sublethal levels of supersaturation may also reduce survivability to juvenile salmon after seawater transfer [5]. However, research directly linking long-term sublethal TDG exposure in freshwater to survival after seawater transfer remains limited. Further studies are needed to fully understand the long-term ecological impacts of TDG on salmonid populations transitioning to marine environments.

There are both inter- and intraspecific differences among Pacific salmonids in their susceptibility to TDGS [15,30]. According to White et al. [31], juvenile brown trout are more sensitive to TDGS than rainbow trout (*Oncorhynchus mykiss*), suggesting differences in the tolerance between Pacific salmonids (*Oncorhynchus* spp.) and Atlantic salmonids. However, tolerances of European species of freshwater fish, such as Atlantic salmon, brown trout, and European Minnow (*Phoxinus phoxinus*), to TDGS and the development of GBD are poorly documented. Most experiments on salmonids have been performed on parr, and we lack knowledge whether the effects differ among life stages and on recovery after GBD. Fish tolerance to TDGS varies across life stages, with a general decline in tolerance as juvenile salmonids grow in age and size. Specifically, alevins and fry tend to be more tolerant to TDGS than older stages, such as parr and smolt [5,8,26].

There are currently no legal regulations or guidelines for supersaturation in Norwegian or European water bodies. Species-specific and life stage specific knowledge are needed to guide management decisions regarding potential regulations. Furthermore, non-salmonid fish, such as European minnow (Cypriniformes) is widespread in south-eastern Norway and often share the same habitat as salmonids. Previous studies have shown that cyprinids are more tolerant to TDGS than salmonids [30]. However, these studies do not include European minnow or other non-salmonid freshwater fish in Europe with exception of carp (Cypriniformes: *Cyprinius carpio)* and the invasive species black bullhead (Siluriformes: *Ameiurus melas*).

The main objective of the present study was to investigate juvenile Atlantic salmon, brown trout, and European minnow for the effects of gas supersaturation on various levels of TDGS over time under controlled laboratory settings. The study aimed to identify clinical signs of GBD and gross pathology across different fish species and life stages during exposure to varying levels of gas supersaturation. Additionally, we aimed to determine the lethal TDG level and explore the potential for fish recovery from GBD.

## Materials and methods

Supersaturated water was generated by mixing atmospheric air with water with increasing pressure inside a 1.5 m cone-shaped column. Supersaturated water stabilized at TDG 140% was then mixed with saturated water in four tanks to generate the treatment levels of supersaturation (TDG 105%, 110%, 115%, and 120%). Dissolved oxygen and TDG levels were logged continuously using a Weiss Saturometer, GhPa-400 Fisch- und Wassertechnik, precision ± 10 hPa (approx. ± 1%), every 30 minutes during the trial periods [18], followed by daily manual control measurements (portable TGP meter; Point Four^TM^ Tracker) of TDG levels in the fish tanks. Measurements also included a control group with untreated water (TDG 100%). The saturometers were pre-calibrated by the manufacturers and recalibrated at atmospheric pressure before application. See also Velle et al. [21] for details on the experimental setup.

The fish tanks used in the trials measured 1 x 1 m with a water depth of 30 cm (300 L). The depth was chosen to limit compensation depth, to enable comparisons to other studies, and represents water depths that the species involved often use. The water depth provides a maximum hydrostatic compensation of 22 mm Hg (about 3% TDG). Fish were acclimated for one week in a stock tank prior to transfer to the trial tanks. Transfer of fish to treatment tanks was done randomly to avoid differences in fish size among groups. The fish were reared under the 12:12 light-dark interval with daily feeding during the study. In addition to manual control measurements of TDG levels, temperatures and oxygen were measured as daily routine in every treatment tank during the trials. Careful adjustments to the water parameters were made whenever measurements deviated by ±0.5 units. Behaviour of the fish were noted daily or several times a day when acute GBD was suspected. Behaviour was observed visually from the surface or via camera (video clips). A scoring scheme was developed and used to describe vertical distribution of the fish in the tanks and the swimming activity, which included response to external stimuli (flight response triggered by sudden water surface disturbance or in connection with the collection of fish for sampling and diagnostics) and loss of equilibrium. Swimming behaviour was recorded as an early-warning, non-diagnostic measure. Given species- and stage-specific variability and tank effects, behaviour alone was not used to diagnose GBD.

The setup was employed in four distinct experimental trials involving: 1. Atlantic salmon parr, 2. Atlantic salmon alevin and fry, 3. Brown trout parr including parr of Atlantic salmon and rainbow trout used as references groups, and 4. Adult European minnow. All fish were quarantined for one week in tanks maintained at 100% TDG, with the same temperature and flow rate as the trial tanks (acclimation), prior to the start of the trials. Routine monitoring of fish health status was conducted before the commencement of the trials, with no detected or suspected infectious diseases, as indicated by normal behaviour and feeding activity. Salmonids originated from tank-reared facilities, while European minnows were wild-caught.

### Ethics statement, animal welfare and human endpoint (HEP)

All trials were conducted at the Industrial and Aquatic Laboratory (ILAB) in Bergen, Norway. ILAB is certified under the ISO 9001:2015 standard and operates in facilities approved by the Norwegian Food Safety Authority. As a member of Norecopa (www.norecopa.no), ILAB is committed to conducting animal experiments in accordance with the 3Rs principles and the objectives of EU Directive 2010/63/EU on the protection of animals used for scientific purposes. ILAB has its own animal welfare body, trained staff in laboratory animal science, and aquamedicine biologists who monitor fish health, safety, and welfare in all ongoing experiments, ensuring compliance with Norwegian laws and regulations on animal experimentation. The experimental period for fish exposed to TDGS was set to a maximum of 14 days. Throughout this period, the fish were handled with care and observed for extended periods each day. Before examination and sampling, all fish were euthanized using a lethal dose of buffered tricaine methanesulfonate (Finquel® vet.). The euthanasia solution was prepared by mixing 5 litres of fish-tank water with Finquel® vet. at a concentration of 600 mg L ⁻ ¹ for salmonid parr and minnows, and 1000 mg L ⁻ ¹ for salmon larvae (alevin and fry). An equal amount of sodium bicarbonate was added to buffer the solution and maintain a neutral pH of 7.

Groups were terminated (fish euthanized) immediately at the human endpoint (HEP). To minimize suffering, clear HEPs were established for all fish exposed to TDGS. Fish were continuously monitored during routine husbandry for abnormal swimming behaviour (e.g., loss of equilibrium, inability to maintain position in the water column) and moribund state (no response to gentle stimulation). Fish exhibiting severe or moribund signs were euthanized immediately using an overdose of anaesthetic (Finquel® vet. as described above) in accordance with the Norwegian Animal Welfare Act (LOV-2009-06-19-97). These criteria were applied consistently across all species and life stages to minimize stress and mortality while ensuring collection of relevant experimental data. A lethal threshold, defined as the point at which 50% of the fish in a group had died or become moribund (LT_50_), was also used as HEP in our study.

The protocols and procedures for the experimental trials in our study were ethically reviewed and approved by the Norwegian Food Safety Authority (FOTS ID: 13176, FOTS ID: 22301).

### Trial 1 – Atlantic salmon (*Salmo salar*)

The trial included cultivated mixed-sex Atlantic salmon parr from the Stofnfiskur strain (Iceland). The trial included five groups assigned to the DGS treatment levels 100% (control), 105%, 110%, 115%, and 120% TDG. Three fish tanks per group were used with 20 fish per tank adding up to 15 tanks and 300 fish. Mean fish size ± standard deviation at startup was 43.10 ± 4.79 g and 15.70 ± 0.63 cm (n = 60). Water flow was set to 2200 litres per hour and with water temperature of 6 °C to emulate spring temperatures when supersaturation often occurs. Treatment time was planned to be 14 days of exposure or until severe clinical signs of GBD occurred. The experiment was conducted from 22 November to 4 December 2017. Trial 1 was carried out as a pre-project and the set-up differs from the subsequent trials in using higher water flow rate and lower water temperature. The water flow rate and temperature in trials 2–4 were adjusted to better fit other stages of Atlantic salmon and other fish species.

### Trial 2 – Atlantic salmon alevins and fry (*Salmo salar*)

Trial 2 included a) alevin (yolk sac larvae) and b) fry of Atlantic salmon, both from the Stofnfiskur strain (Iceland). The trial was set up with DGS treatments at 100% (control), 105%, 110%, 115%, and 120% TDG. Each group was set up with duplicate tanks, containing 25 alevins and 25 fry each. Alevins were placed in 30 x 30 cm open boxes with a substrate of astroturf to separate them from the fry. A total of 250 alevins and 250 fry were used in trial 2. Mean fish size ± standard deviation at start-up was 0.13 ± 0.04 g and 2.68 ± 0.09 cm for alevins (n = 50), and 0.26 ± 0.05 g and 3.14 ± 0.09 cm for fry (n = 50). Water flow was set to 500 litres per hour with a water temperature of 8 °C. The treatment period was set to 14 days of exposure. Trial 2 also included a 4-day recovery trial using 100% TDG for all groups. Experiments were performed from 26 February to 13 March 2020.

### Trial 3 – Brown trout (*Salmo trutta*), Atlantic salmon, and rainbow trout (*Oncorhynchus mykiss*)

Experiment 3 used tank-reared brown trout parr with wild caught parent fish from Eidfjord (SIMA hatchery, Statkraft). The tank set up was the same as in trial 2, featuring five groups in duplicate tanks. A total of 340 brown trout parr were used in trial 3. Mean fish size ± standard deviation at startup was 23.02 ± 6.49 g and 12.96 ± 1.21 cm (n = 50). Water flow was set to 400 litres hour^-1^ and water temperature 8 °C with a treatment period of 14 days of exposure. The treatment period in trial 3 was followed by a one-week recovery trial using 100% TDG in all groups. The trial lasted from 16 April to 8 May 2020.

Reference groups using Atlantic salmon parr (the same strain as in trial 1) and rainbow trout parr (hatchery reared strain at ILAB) for treatment level 120% TDG were included in trial 3. These reference groups were included to test reproducibility in the trials and for comparisons of tolerance among different salmonid species of similar size and stages. For this purpose, we included one tank with salmon (n = 25) and one tank with rainbow trout (n = 25) using the treatment group T120 after the brown trout group was terminated. Mean fish size prior to startup was 32.5 g and 14.1 cm (n = 5) for salmon and 34.2 g and 13.8 cm (n = 5) for rainbow trout.

### Trial 4 – European minnow (*Phoxinus phoxinus*)

Trial 4 included wild caught European minnow from Otra river in Southern Norway. The fish were caught by electrofishing and transported in oxygenated water tanks to the laboratory. The trial set up for minnows was the same as in trials 2 and 3 using duplicate tanks (2 x 25 fish), but with no recovery trial. A total of 250 European minnow were used in trial 4. Mean fish size ± standard deviation measured at startup was 1.77 ± 0.93 g and 5.68 ± 0.86 cm (n = 50). Water flow was set to 400 litres hour^-1^ with a water temperature of 8 °C. The experiment was performed from 11 May to 25 May 2020.

### Sampling and diagnostics

Procedure of sampling and diagnostics (gross pathology) followed methods from Mesa et al. [27]. Only live or moribund fish were collected from their tanks and euthanized with a lethal dose of buffered tricaine methanesulfonate solution prior to examination (see *Ethics statement, animal welfare and humane endpoint*). The fish were measured for total body weight (g) and total length (cm) and were immediately scanned for subcutaneous emphysema (gas bubbles in skin, lateral line, fins, and buccal cavity) and gill emboli using a stereo microscope (zoom range 7.5 - 60X). Gill arches were removed and placed in a water-filled petri dish prior to examination. Occurrences of gas embolism in gill samples were noted as positive or negative without further quantification. The final step in the fish examination involved an autopsy to determine gross signs of GBD in the abdominal cavity and gastrointestinal tract.

The occurrence of gas bubbles in the lateral line and fins is given as the prevalence of observation, while the intensity is measured as ranked severity based on percent surface area covered by bubbles [27] using categorized data. Severity is ranked as 0 = negative, no observed bubbles; 1 = less than 25% covered with bubbles; 2 = 26–50% covered; 3 = more than 50% covered. Individual fish condition factor was calculated as Fulton’s K (for K = Weight/Length^3^, given as g cm^-3^).

Moribund fish were recognized as weakened with complete loss of equilibrium and without response to stimuli. Fish in this condition were removed from the tank, euthanized, and examined immediately for clinical signs of GBD.

Development of clinical signs and increased mortality or moribundity within hours of exposure to DGS was termed acute GBD, while prolonged development over days of exposure to DGS was termed subacute GBD.

### Definitions and equations

Total gas pressure (TGP) in water is the sum of the partial pressure of all dissolved gases (∆P = pN_2_ + pO_2_ + pCO_2_ + pH_2_O; mmHg) and barometric pressure (BP; mm Hg). Levels of saturation are commonly termed as total dissolved gas (TDG%). The water is gas supersaturated when ∆P > 0 or TDG > 100% (Colt [2]; see equation 1 and 2).


TGP=ΔP+BP (1)

TDG%=100 (ΔP+BP)/BP, or 100 (TGP/BP) (2)

The water’s capacity to dissolve gas increases with water depth due to hydrostatic pressure. GBD in aquatic animals, such as fish, may occur if the TDG pressure in water is greater than the TDG (barometric + hydrostatic pressure) at the fish position in the water column [2]. Hence, bubble formation in circulatory system and body tissue might be prevented if the fish can seek deeper water, increasing the hydrostatic pressure to compress the gases. The hydrostatic compensation depth depends on the ratio of differential pressure (∆P; mm Hg) and hydrostatic pressure, as shown in Eq. 3.


Z=ΔP/ 73.42, for Z=hydrostatic compensation depth (meter) (3)

The factor 73.42 represents hydrostatic pressure in freshwater expressed as mm Hg m^-1^ [32]. That is, TDG decreases with 73.42 mm Hg per meter water depth. TGP and TDG% corrected for water depth is shown in Eq. 4.


$$\begin{array}{cc} {\text{TGP}}_{\text{corrected}}=\; & (\Delta \text{P}+\text{BP})/(\text{BP}+\text{pd})\Rightarrow {\text{TDG}}_{\text{corrected}}\%=\text{100}\;({\text{TGP}}_{\text{corrected}}/\;\text{BP}),\;\\ & \text{for}\;\text{p}=\text{hydrostatic}\;\text{pressure}\;(\text{73}.\text{42}\;\text{mm}\;\text{Hg})\;\text{and}\;\text{d}=\text{water}\;\text{depth}\;(\text{m}) \end{array}$$ (4)

### Numerical analyses

Analysis of variance (one-way ANOVA) assuming homogeneity of variance and normality (Levene’s test and Shapiro-Wilk W, respectively, for p > 0.05) was used to compare variations in water parameters (temperature, TDG) and fish size between replicate fish tanks and between groups in the different trials. Tukey HSD was used as a post hoc test in case of significant ANOVA result (p < 0.05). Student-t test was used to compare variations between duplicate tanks. When assumptions for parametric testing were not met, non-parametric alternatives were applied, including the Kruskal–Wallis ANOVA followed by multiple comparisons of z’-values (equivalent to Dunn’s post hoc test), as well as Mann–Whitney U tests. A Kolmogorov-Smirnov analysis was applied to compare ranked severity of GBD to fish in the treatment groups.

Frequency of observations among groups (signs present or absent) were compared with use of a 2 x 2 contingency table and Fisher’s Exact Test. Differences in observed clinical signs of GBD between fish in control and treatment groups were determined using Fisher’s exact test (FET) rather than the Chi-square (χ^2^) due to low sample size (n ≤ 60). The Bonferroni correction was applied to adjust the significance level for as control for multiple comparisons, using the formula: α _adjusted _= α/ *m*, where α is the original significance level (0.05) and *m* is the number of pairwise comparisons.

Temporal variations in water quality and Fulton’s K-factor were determined using the non-parametric Kendall’s Tau rank correlation.

Mortality and observed subcutaneous emphysema (gas bubbles in fins) among groups were plotted as cumulative percentage during the experimental trial period. Cumulative mortality or the presence of gas bubbles in the fins of a group was calculated as the ratio of the total number of observed cases (dead or moribund fish, or fish with gas bubbles) during a given exposure period to the initial population size of the group. Frequent monitoring (hourly or daily) of dead or moribund fish counts were conducted to track mortality progression. Final mortality proportions were analysed using Fisher Exact test.

Temporal development of subcutaneous emphysema in fish (rates of bubble formation) exposed to different DGS levels was determined with use of linear regression and statistical tests to examine differences of the slopes among treatments.

Statistical analyses were performed with use of the software Statistica® 13, where p-values <0.05 were considered statistically significant.

## Results

### Water quality

#### Temperature.

Water temperatures in trial 1 ranged from 5.8 °C to 6.3 °C (mean 6.1 ± 0.1) in all treatment tanks. The water temperature in trials 2–4 was adjusted to accommodate other species and stages and was set to 8 °C. The temperature ranged from 7.7 °C to 8.8 °C (mean 8.3 ± 0.2) in trial 2, 7.6 °C to 8.2 °C (mean 7.9 ± 0.1) in trial 3, and 7.8 °C to 8.8 °C (mean 8.3 ± 0.3) in trial 4. There were insignificant differences in water temperatures between tanks and groups across the trials, but water temperature was on average c. 0.4 °C lower in trial 3 (brown trout) compared to trial 2 (salmon alevin and fry) and 4 (minnow). Post-hoc comparisons (z’ values) revealed that temperature in trial 3 differed significantly (p < 0.0001) from trial 2 and trial 4. Insignificant difference were found between trial 2 and trial 4. Mean water temperatures and flow rates in the different groups and trials are given in Table 1.

**Table 1:** Water quality in groups from all trials.

	Groups	T120	T115	T110	T105	T100
**TRIAL 1 –** Salmon parr (Nov. 22nd - Dec. 4th 2017)	Treatment time (days)	1	2	10	13	13
	Temperature (°C)	5.9	6.1	6.2 ± 0.1	6.2 ± 0.1	6.1 ± 0.1
	Flow rate	2200	2200	2200	2200	2200
	TDG level (%)	118-121	115-118	108.9 ± 1.0	105.2 ± 0.8	100.6 ± 0.2
	TDG_corr_ (%)	115-118	112-115	106	102	98
	ΔP (mm Hg)	138	107	66	39	4.5
	BP (mm Hg)	735	733.3	743.8 ± 8.7	746.8 ± 9.5	746.8 ± 9.5
	O_2_ (%)	126.8	115.6	108.3	100.4	99.5
	N_2_ (%)	116.9	114.5	109.2	106.5	100.9
**TRIAL 2** – Salmon alevins and fry (Feb. 26th - Mar. 13th 2020)	Treatment time (days)	14	14	14	14	14
	Temperature (°C)	8.2 ± 0.2	8.3 ± 0.2	8.3 ± 0.2	8.2 ± 0.2	8.3 ± 0.2
	Flow rate	500	500	500	500	500
	TDG level (%)	120.6 ± 0.5	115.8 ± 0.7	109.9 ± 0.8	106.1 ± 0.3	99.7 ± 0.9
	TDG_corr_ (%)	116	112	107	103	97
	ΔP (mm Hg)	145	114	73	45	−2
	BP (mm Hg)	741.0 ± 6.4	741.0 ± 6.4	741.0 ± 6.4	741.0 ± 6.4	741.0 ± 6.4
	O_2_ (%)	114.9	111.6	108.4	104.5	100.9
	N_2_ (%)	122.4	117.0	111.6	105.7	99.5
**TRIAL 3** – Brown trout parr (Apr. 16th - May 7th 2020)	Treatment time (days)	1	3	14	14	14
	Temperature (°C)	7.8 ± 0.1	7.6 ± 0.1	7.9 ± 0.1	7.9 ± 0.2	7.9 ± 0.1
	Flow rate	400	400	400	400	400
	TDG level (%)	121.1 ± 1.0	115.3 ± 0.4	110.7 ± 1.2	105.7 ± 0.4	101.1 ± 1.0
	TDG_corr_ (%)	118	112	108	103	98
	ΔP (mm Hg)	160	116	81	43	8
	BP (mm Hg)	757.5 ± 2.5	760.6 ± 3.4	757.7 ± 7.9	757.7 ± 7.9	757.7 ± 7.9
	O_2_ (%)	115.9	112.5	107.6	102.9	98.8
	N_2_ (%)	120.2	115.7	110.9	105.5	101.7
**TRIAL 4** – European minnow (May 11th - May 25th 2020)	Treatment time (days)	14	14	14	14	14
	Temperature (°C)	8.2 ± 0.4	8.2 ± 0.4	8.3 ± 0.1	8.4 ± 0.1	8.2 ± 0.2
	Flow rate	400	400	400	400	400
	TDG level (%)	119.4 ± 2.0	114.7 ± 1.3	109.4 ± 1.3	106.3 ± 0.4	100.2 ± 0.6
	TDG_corr_ (%)	116	111	106	103	97
	ΔP (mm Hg)	147	111	71	48	2
	BP (mm Hg)	757.2 ± 7.2	757.2 ± 7.2	757.2 ± 7.2	757.2 ± 7.2	757.2 ± 7.2
	O_2_ (%)	118.2	112.9	108.7	104.3	100.1
	N_2_ (%)	120.0	115.4	110.9	106.0	100.2

Trial 1 was carried out in 2017 and trials 2–4 in 2020. Daily measurements of temperature and TDG are given as mean±SD or range (min-max). Flow rate is given as litre per hour. Oxygen and nitrogen saturation (%) is given as mean. ΔP corresponds to the mean or measured TDG level. TDG_corr_ is the mean TDG corrected for 30 cm compensation depth in the fish tanks and barometric pressure. Barometric pressure (BP, mm Hg) was obtained from the Geophysical Institute at University of Bergen, Norway (https://veret.gfi.uib.no/).

#### Measurements of total dissolved gas (TDG).

**Fig 1:**
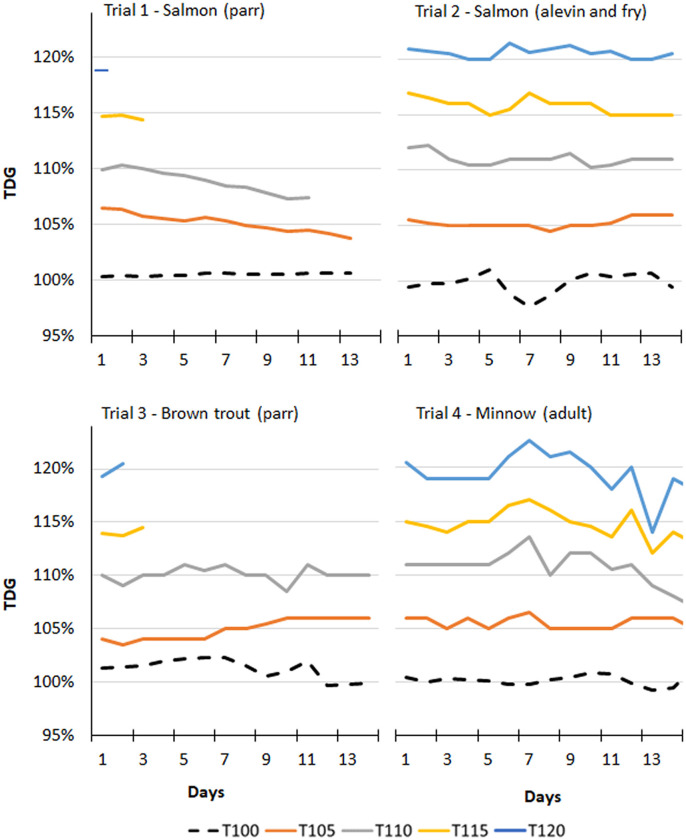
Measurements of total dissolved gas supersaturation. Daily mean total dissolved gas (TDG) supersaturation measured in fish tanks in treatment groups T100 (100% TDG, control), T105 (105% TDG), T110 (110% TDG), T115 (115% TDG), and T120 (120% TDG). There were four trials (1-4) including Atlantic salmon (*Salmo salar*), brown trout (*Salmo trutta*), and European minnow (*Phoxinus phoxinus*). Groups T115 in trial 1, T120 in trial 3, and T110 in trial 1 were terminated after 48 hours, 24 hours, and 10 days, respectively, due to high fish mortality or severe clinical signs of gas bubble disease.

Daily TDG measurements in the fish tanks showed insignificant difference among replicate tanks within groups in trials 1–4 during treatment periods (t-tests, p > 0.05). However, continuous and daily control measurements indicated temporal variations in TDG within groups across trials, which may have influenced the results (Fig 1).

In trial 1, there were temporal changes in TDG in both T105 and T110. TDG decreased from 106.5% to 103.8% (Kendall’s τ = −0.92, p < 0.0001) during 13 days of measurements in T105, and from 110.4% to 107.4% (Kendall’s τ = −0.91, p < 0.001) over a period of 10 days in T110. TDG in T100 (control) was most stable and varied from 100.4% to 100.7% during a period of 12 days. Groups T115 and T120 in trial 1 were terminated within 24 hours due to high fish mortality, hence, no long-term temporal changes in TDG were available from these groups. TDG ranged from 118% to 121% in T120 and from 115% to 118% in T115.

In trial 2, highest variance of TDG was measured in T100 (control) with values ranging from 97% to 101%. The TDG in T115 decreased from 117% to 115% during a period of 14 days (Kendall’s τ = −0.58, p < 0.001).

In trial 3, TDG in T105 increased from 104% to 106% during 14 days of measurements (Kendall’s τ = 0.83, p < 0.0001). Temporal variations in TDG were most prominent in Trial 4, especially in groups T120 (114% − 123%), T115 (112% − 117%) and T110 (107% − 114%). However, TDG decreases in all groups in trial 4 were not statistically significant for p < 0.05.

### Fish size

A summary of the biotic details describing fish sizes and K-factors from the experimental trials 1–4 is given in Table 2. Comparisons of fish size and K-factor for salmonid parr at the onset of trial 1 and 3 is shown in Table 3.

**Table 2:** Biotic details from the experimental trials 1-4.

	Groups	T120	T115	T110	T105	T100
**TRIAL 1** – Salmon	Exposure time	4 hrs	24 hrs	10 days	13 days	13 days
	No. sampling	1	2	4	4	4
	Total N	60	60	60	60	60
	Sampling N	15	30	59	60	60
	Total length (cm)	15.5 ± 0.5	15.7 ± 0.5	15.7 ± 0.6	15.7 ± 0.6	15.7 ± 0.7
	Weigth (g)	42.9 ± 3.9	42.7 ± 3.9	42.5 ± 4.8	42.2 ± 4.8	42.7 ± 5.1
	K-factor	1.15 ± 0.05	1.11 ± 0.04	1.11 ± 0.07	1.09 ± 0.05	1.11 ± 0.06
**TRIAL 2a** - Alevin	Exposure time	14 days†	14 days†	14 days†	14 days†	14 days†
	No. Sampling	3 (2)	3 (2)	3 (2)	3 (2)	3 (2)
	Total N	50	50	50	50	50
	Sampling N	50	49	50	50	49
	Total length (cm)	2.81 ± 0.12	2.80 ± 0.12	2.76 ± 0.09	2.79 ± 0.11	2.73 ± 0.14
	Weight (g)	0.16 ± 0.03	0.16 ± 0.03	0.16 ± 0.03	0.16 ± 0.03	0.14 ± 0.04
	K-factor	0.73 ± 0.13	0.71 ± 0.13	0.78 ± 0.16	0.74 ± 0.12	0.68 ± 0.16
**TRIAL 2b** - Fry	Exposure time	14 days†	14 days†	14 days†	14 days†	14 days†
	No. Sampling	4 (1)	3 (2)	3 (2)	3 (2)	3 (2)
	Total N	50	50	50	50	50
	Sampling N	50	50	50	50	50
	Total length (cm)	3.29 ± 0.16	3.36 ± 0.18	3.39 ± 0.18	3.40 ± 0.21	3.33 ± 0.24
	Weight (g)	0.31 ± 0.05	0.33 ± 0.07	0.35 ± 0.05	0.35 ± 0.09	0.31 ± 0.08
	K-factor	0.87 ± 0.11	0.87 ± 0.12	0.89 ± 0.08	0.86 ± 0.14	0.83 ± 0.11
**TRIAL 3** – Brown trout	Exposure time	13 hrs	48 hrs	14 days‡	14 days‡	14 days‡
	No. Sampling	1	2	5 (1)	3 (1)	3 (1)
	Total N	50	50	80	80	80
	Sampling N	30	38	79	70	70
	Total length (cm)	13.3 ± 1.13	13.5 ± 1.0	12.7 ± 1.4	13.3 ± 1.3	13.4 ± 1.3
	Weight (g)	25.1 ± 7.3	25.97 ± 6.5	21.3 ± 7.6	24.9 ± 8.0	25.0 ± 8.0
	K-factor	1.04 ± 0.07	1.03 ± 0.07	0.99 ± 0.06	1.02 ± 0.08	1.01 ± 0.08
**TRIAL 4** – Minnow	Exposure time	14 days	14 days	14 days	14 days	14 days
	No. Sampling	3	3	3	3	3
	Total N	50	50	50	50	50
	Sampling N	36	33	36	34	31
	Total length (cm)	5.61 ± 1.02	5.81 ± 1.00	5.55 ± 0.94	5.49 ± 0.80	5.69 ± 0.87
	Weight (g)	1.85 ± 0.99	1.96 ± 1.22	1.66 ± 0.94	1.57 ± 0.81	1.74 ± 0.87
	K-factor	0.93 ± 0.11	0.90 ± 0.13	0.88 ± 0.13	0.87 ± 0.11	0.87 ± 0.10

Trial 1: Atlantic salmon parr (*Salmo salar*); Trial 2a: Atlantic salmon alevin; Trial 2b: Atlantic salmon fry; Trial 3: Brown trout parr (*S. trutta*); Trial 4: European minnow adults (*Phoxinus phoxinus*). Experimental periods are provided in brackets. Different groups (T100 - T120) were exposed to varying levels of gas supersaturation ranging from 100% TDG (control) to 120% TDG. The treatment periods for trials 2 and 3 included a recovery period of 3 days (†) or 7 days (‡) with TDG levels normalized across all groups (TDG 100%). Number of samplings for trials 2 and 3 in the extended period are specified in brackets. A group was terminated when more than 50% of the fish became moribund or died. Exposure times are listed in hours or days. Fish size and Fulton’s K are presented as mean ± SD.

**Table 3:** Fish size and Fulton’s K for salmonid parr at the onset of trials 1 and 3.

	Salmon (trial 1)	Brown trout (trial 3)	Salmon (trial 3)	Rainbow trout (trial 3)
	n = 90	n = 22	n = 17	n = 6
**Length (cm)**	15.6 ± 0.6 (15.6)*	13.1 ± 1.1 (13.0)	14.4 ± 0.5 (14.4)	13.6 ± 0.38 (13.6)
	14 - 17	11 - 15	13 - 16	13–15
**Weight (g)**	42.9 ± 4.6 (43.0)*	23.4 ± 6.6 (22.0)	35.3 ± 3.5 (35.8)	31.9 ± 2.2 (31.3)
	33 - 54	16 - 38	26 - 43	29–36
**K**	1.11 ± 0.05 (1.11)*	1.02 ± 0.07 (1.00)*	1.17 ± 0.04 (1.17)	1.28 ± 0.06 (1.27)
	0.95 - 1.30	0.88 - 1.13	1.07 - 1.27	1.22 - 1.40

Length, weight and Fulton’s K are given as mean ± SD (median) and range for salmon (*Salmo salar*), brown trout (*S. trutta*) and rainbow trout (*Oncorhynchus mykiss*). Statistic significant differences (p < 0.05) are marked with asterisk (*).

#### Trial 1: Atlantic salmon (total n = 300 parr).

There was no statistically significant difference in mean fish size (weight or length) among the treatment tanks and groups (T100, T105, T110, T115, T120: one-way ANOVA, p > 0,05) at the onset or end of sampling in this trial. Groups T120 and T115 were terminated within 24 hours due to high mortality. Fish with prolonged exposure included T100 (control group), T105 and T110. Fulton’s K among fish in T110 decreased over time until termination after ten days (Kendall’s τ = −0.22, p < 0.02), however, mean K to fish in T110 was not significantly different from fish in the control group (T100) at termination (t-test; p = 0.28). There were no significant temporal changes in K-factor among fish in T105 or in the control group (T100) during the 13 days exposure time.

#### Trial 2: Atlantic salmon alevin (total n = 250) and fry (total n=250).

There were no statistically significant differences in alevin size (weight or length) among the groups at the onset of the trial, but fish from T100 (control) was larger (both length and weight) than fish from the other groups in the last sampling after 17 days (inclusive 3 days recovery). Weight to alevins from T100 (control) were significantly higher than alevins from T115 (p = 0.003) and T120 (p = 0.0001) in the last sampling (Multiple comparisons of z’ values). There were no statistically significant differences among Atlantic salmon fry size in the groups at the onset or end of the trial.

#### Trial 3: Brown trout (total n = 340 parr).

There was insignificant difference in fish size or Fulton’s K between the control group (T100) and the other groups at the onset of this trial. Groups T115 and T120 were terminated within 48 hours due to high mortalities. Hence, fish with prolonged exposure included T100 (control), T105 and T110. Only fish from the control group (T100) showed a significant increase in growth (weight, length) during the 21 days trial (Kendall’s τ > 0.18, p < 0.03). Fish from control group T100 were significantly larger (n = 40; mean weight 26.4 ± 8.2 g, mean length 13.6 ± 1.3 cm) than fish from group T110 (n = 40; mean weight 22.1 ± 7.7 g, mean length 12.9 ± 1.4 cm) in the last samplings after 14 and 21 days (t-test, p < 0.02).

#### Atlantic salmon (total n = 25 parr) and rainbow trout (total n = 25 parr).

Two additional groups including Atlantic salmon parr (n = 25) and rainbow trout parr (n = 25) were included in trial 3 for treatments with TDG 120%. Trial 3 aimed to use fish of a similar development stage and size as in trial 1. However, the results indicate that trial 1 Atlantic salmon were significantly larger than salmonids in trial 3 (Multiple comparisons z’ values; p < 0.001; Table 3).

#### Trial 4: European minnow (total n = 250 adults).

There was insignificant difference in fish size (weight and length) or Fulton’s K among minnows among the treatment groups (T100, T105, T110, T115 and T120) at the onset of the trial or at the end of the trial.

### Clinical signs of gas bubble disease

There was insignificant difference (FET; p > 0.05) in the prevalence of observed clinical signs of GBD among fish sampled from replicate tanks within a group (T100, T105, T110, T115, T120) in any of the trials. However, there were significant difference in the development of GBD among the groups.

#### Atlantic salmon parr (trial 1).

**Fig 2:**
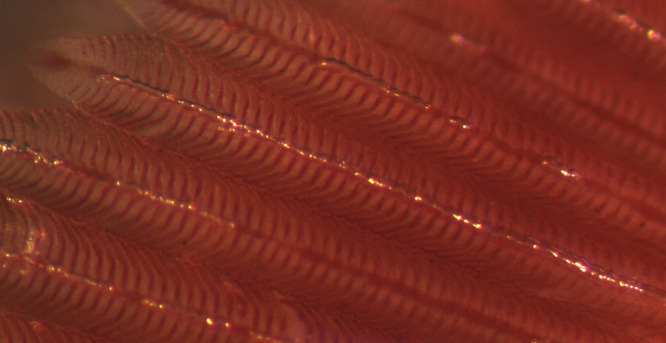
Gas embolism in gills. Clinical signs of gas bubble disease in Atlantic salmon (*Salmo salar*) parr, manifested as gas embolism in the gills.

The acute phase of GBD occurred among Atlantic salmon parr from groups T120 and T115. Clinical signs included gas embolism in the gills, internal bleeding (bloody ascites) and subcutaneous emphysema in the fins. Gas embolism in gills was easily detected with use of stereomicroscope, appearing as intravascular bubbles in the gill filaments (Fig 2). Prevalence of gas embolism in gills increased with TDGS. Significant differences were found between T120 and T110 (FET; p < 0.00001) and between T115 and T110 (FET; p = 0.0042) with significance level adjusted for multiple comparisons. No gas embolism was observed in fish from T105 or T100.

**Fig 3:**
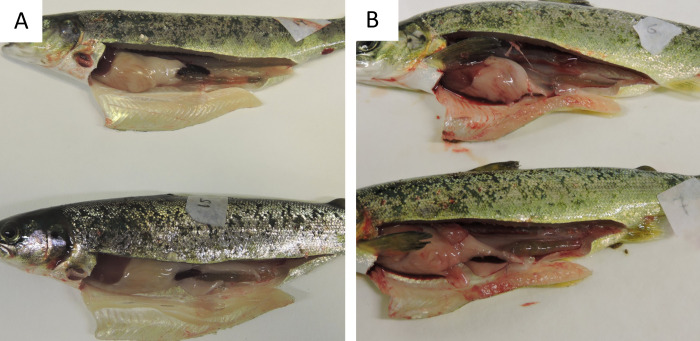
Internal bleeding. Clinical signs of gas bubble disease in Atlantic salmon (*Salmo salar*) parr, manifested as internal bleeding in the abdominal cavity. A: Normal, no signs; B: Internal bleeding visible as haemorrhagic ascites.

Internal bleeding, appearing as hemorrhagic ascites (Fig 3), in the abdominal cavity co-occurred with gas embolism in the gills among fish in the acute phase of GBD (TDG ≥ 115%; FET, p = 0.0003).

**Fig 4:**
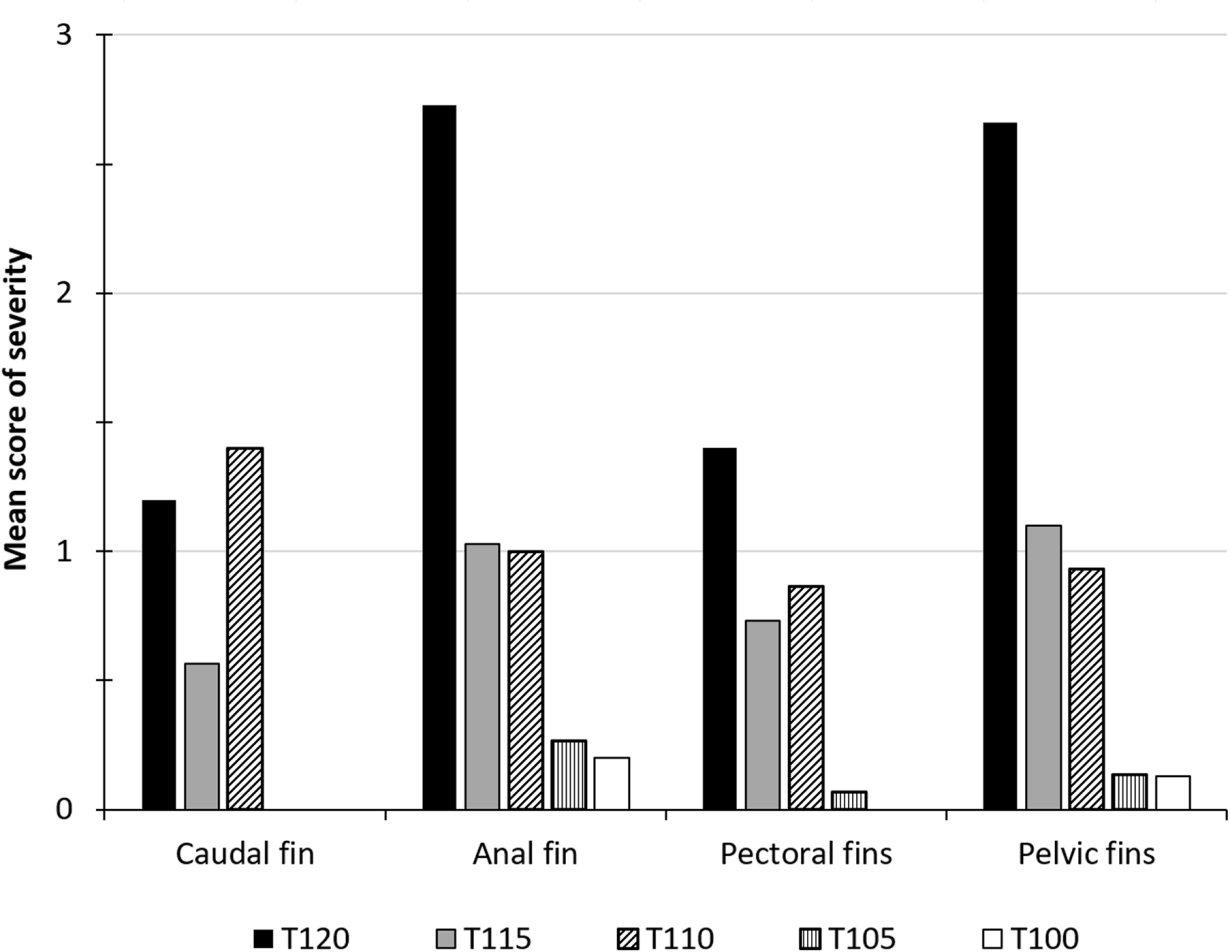
Ranked severity of gas bubble formation in fins of salmon parr exposed to different levels of TDG supersaturation. The mean intensity of subcutaneous emphysema (gas bubbles) in fins of Atlantic salmon (*Salmo salar*) parr scored on a scale that indicate the coverage of bubbles on the fins: 0 = No bubbles oserved, 1 = less than 25% covered, 2 = 26-50%, and 3 = more than 50%. The groups (T) were exposed to total dissolved gas (%) according to the number in their names. T120 (n = 15) was exposed for 3 hours, T115 (n = 30) was exposed for 24 hours, T110 (n = 15) was exposed for 10 days, T105 (n = 15) was exposed for 13 days, and T100 (control, n = 15) was exposed for 13 days.

Subcutaneous emphysema appeared in both unpaired (caudal and anal) and paired fin (pelvic and pectoral) in all examined fish from group T120 within three to four hours of exposure. The highest severity score was observed in anal and pelvic fins (score 2 or 3) in all examined fish from group T120 (Fig 4). Average fin scores (ranked severity) of subcutaneous emphysema in anal fin and pelvic fins were significantly higher in T120 compared to fish from the other groups when observations from the last samplings in the groups were compared (Multiple comparisons of z’ values; p < 0.025). Groups T115 and T120 were terminated one day post-exposure to DGS due to high mortality.

**Fig 5:**
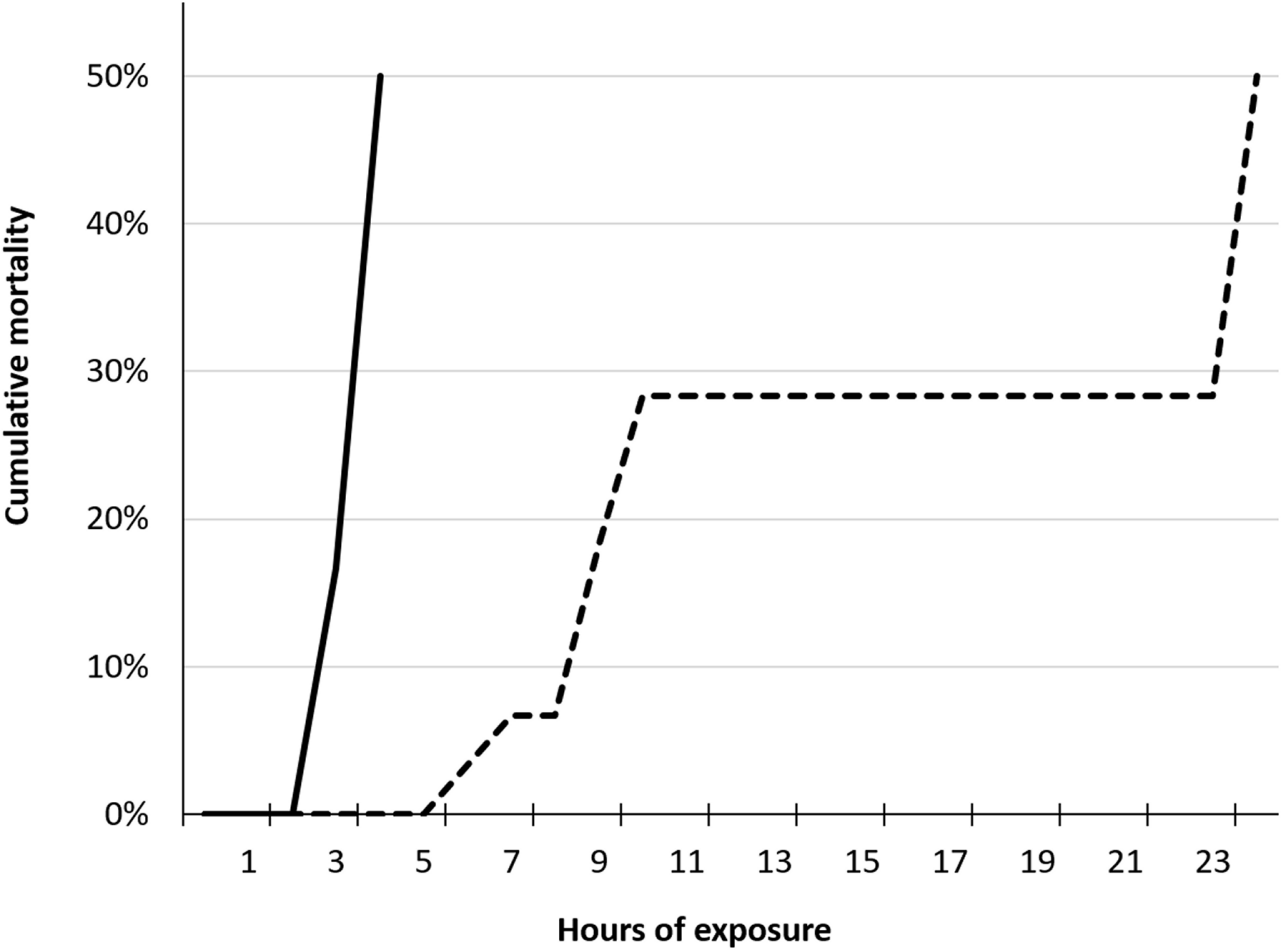
Lethal TDG supersaturation levels for salmon parr during acute gas bubble disease. Cumulative mortality of Atlantic salmon (*Salmo salar*) parr exposed to 115% (dotted line; n = 30) and 120% (solid line; n = 30) total dissolved gas (TDG) supersaturation.

Cumulative mortality of 50% (LT_50_) occurred within four hours among fish in T120 and T115 after 24 hours (Fig 5). Group T110 was terminated after 10 days of exposure due to severity of GBD, while T100 and T105 were terminated after 13 days, as initially planned. Dead fish were not examined for signs of GBD in trial 1.

The observed prevalence and severity of subcutaneous emphysema observed in fish from group T115 after 24 hours of exposure (acute GBD) were not significantly different from the observed signs that appeared subacute among fish from group T110 after more than 6 days of exposure (Kolmogorov-Smirnov two-sample Test, p > 0.05). Overall, long-time exposure of TDG from 107 to 110% (T110) revealed similar signs of GBD as for acute GBD. Internal bleeding was also registered in one fish from group T110 after ten days of exposure (subacute). Additional signs associated with GBD, such as exophthalmia, bubbles in the head region (buccal cavity, operculum), and subcutaneous emphysema in adipose fin, only appeared subacute among fish in T110. The latter subacute signs appeared after a week of exposure to TDG levels ranging from 108% to 111%.

**Fig 6:**
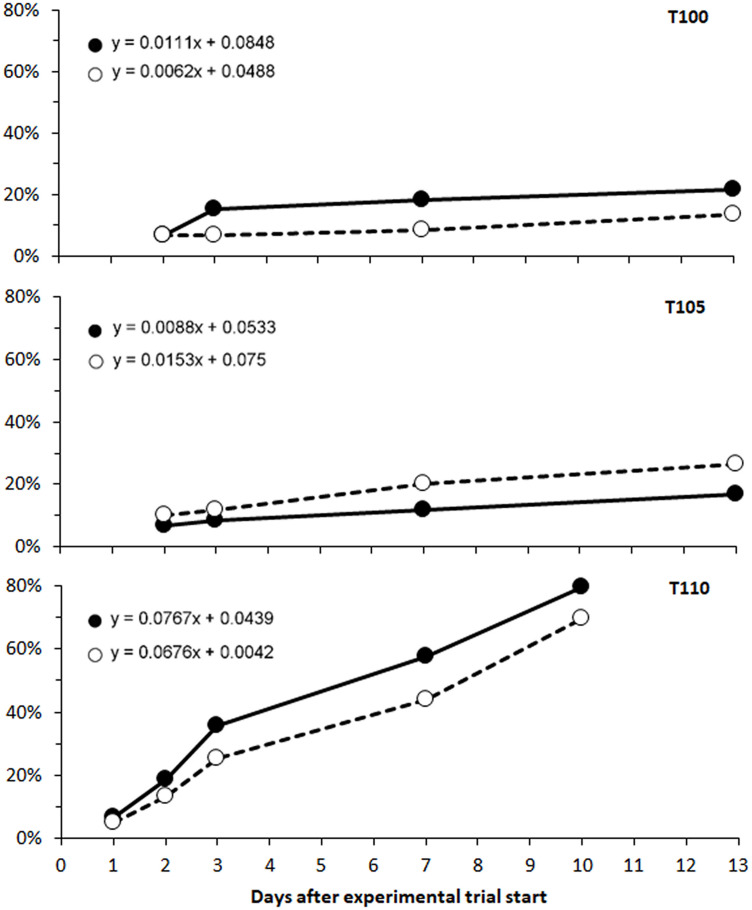
Rates of gas bubble formation in fins to salmon parr during subacute gas bubble disease. Cumulative prevalence of bubble formation in unpaired (○; caudal and anal) and paired (●; pectoral and pelvic) fins in Atlantic salmon (*Salmo salar*) parr exposed to total dissolved gas (TDG) of 100% (T100, control), TDG supersaturation of 105% (T105), and TDG supersaturation of 110% (T110). The formation of bubbles is expressed as a linear regression, where the coefficient of determination (R^2^) was > 0.95 for all exposure groups, except for the paired fins of fish in treatment group T100 (R^2^ = 0.74).

The prevalence of observed emphysema among fish in T110 increased during the exposure time, with a cumulative bubble formation rate calculated as 6.8% and 7.7% per day for unpaired fins (caudal and anal fins) and paired fins (pectoral and pelvic), respectively (Fig 6).

**Fig 7:**
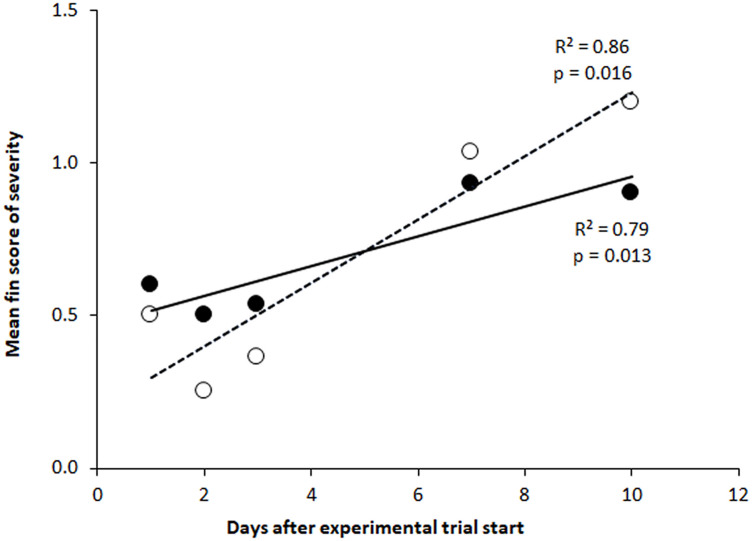
Progress in formation of gas bubbles in fins of salmon parr during subacute gas bubble disease. Mean fin score of severity was measured in fish exposed to 110% total dissolved gas (TDG) supersaturation during a period of 10 days (T110). Observations of gas bubble formations in unpaired fins (○ and dotted line; caudal and anal fins) and paired fins (● and solid line; pectoral and pelvic fins) of Atlantic salmon (*Salmo salar*) parr. N = 5 to 15 fish per dot. Severity of bubble formation ranked on a scale that indicate the coverage of bubbles on the fins: 0 = No bubbles observed, 1 = less than 25% covered, 2 = 26-50%, and 3 = more than 50%. Rates of severity are expressed as a linear regression (y = 0.1037x + 0.1931 for unpaired and y = 0.0485x + 0.4698 for paired). The respective coefficients of determination (R^2^) and p-values are shown in the figure.

The severity of bubble formation also increased significantly over time for the caudal fin (Kendall’s τ = 0.47, p < 0.0001) and pelvic fins (Kendall’s τ = 0.19, p = 0.03). Ranked severity of bubble formations in the unpaired fins (caudal and anal) increased by approximately 10% per day, while severity of bubble formation increased by about 5% per day in the paired fins (pelvic and pectoral; Fig 7).

Subcutaneous emphysema, identified as gas bubbles in the fins, were also observed in a few fish from T105 and T100. The severity was considered low (mild), and the prevalence of fish with bubble formations in fins in T105 and T100 was significantly lower compared to the other groups (FET, p > 0.05). Emphysema in unpaired fins (anal fin) among fish in T105 showed a significantly higher rate of cumulative bubble formation than fish from T100 (control). The prevalence of Atlantic salmon parr with subcutaneous emphysema in the anal fin increased by 1.5% per day when exposed to 105% TDG (Fig 6).

**Fig 8:**
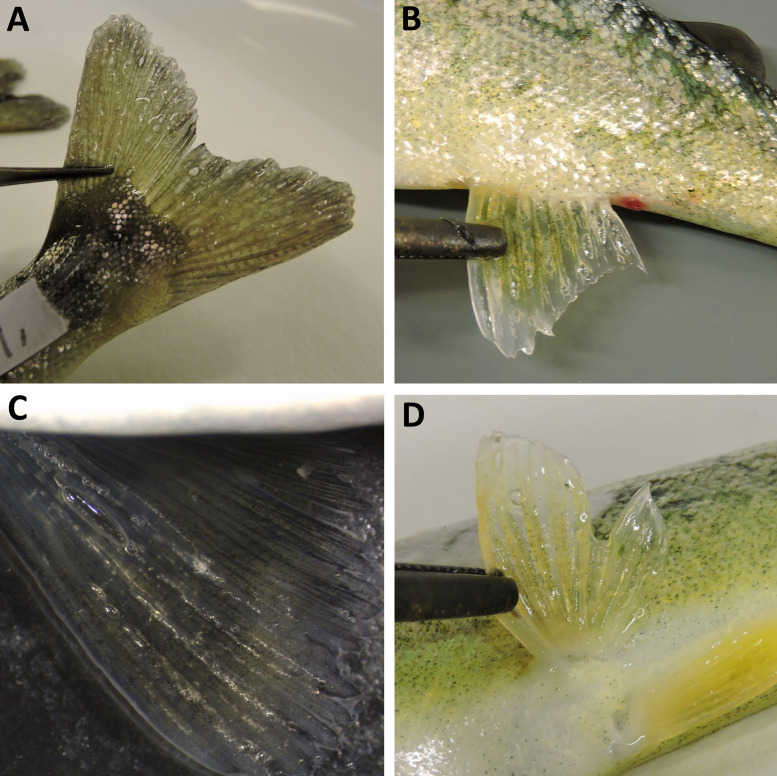
Common signs of acute and subacute gas bubble disease in Atlantic salmon (*Salmo salar*) parr. Gas bubble formations in **(A)** Caudal fins; **(B)** Anal fin; **(C)** Pectoral fin; **(D)** Pelvic fin.

**Fig 9:**
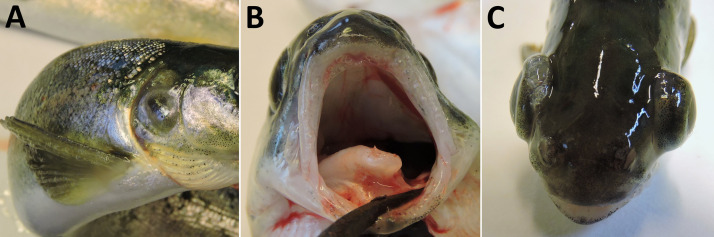
Observed signs of subacute gas bubble disease in Atlantic salmon (*Salmo salar*) parr. **(A)** Gas bubble on operculum; **(B)** Gas bubbles in buccal cavity; **(C)** Bilateral exophthalmia.

Subcutaneous emphysema was also frequently observed in the dorsal fin. However, prevalence and severity of emphysema could not be assessed for all fish due to fin erosion. Dorsal fin erosion occurred among fish in all groups, including T100 (control), and is not considered as an effect of gas supersaturation in this study. We did not observe gas bubbles within the lateral line in the Atlantic salmon parr from the groups in trial 1. Observed clinical signs of GBD among fish in trial 1 are summarised in Table 4 and Figs 8 and 9.

**Table 4:** Clinical signs of gas bubble disease (GBD) in Atlantic salmon parr (*Salmo salar*) from trial 1.

Groups		T120	T115	T110	T105	T100
Exposure time		4 hours	24 hours	10 days	13 days	13 days
No. fish examined		15	30	59	60	60
Mortality		>50%	50%	< 2%	–	–
Gas emboli gills		93%	57%	23%	–	–
Hemorrhagic ascites		80%	23%	2%	–	–
Emphysema	Caudal fin	100%	50%	38%	2%	–
	Dorsal fin	80%	50%	23%	5%	2%
	Anal fin	100%	67%	52%	18%	12%
	Pelvic fin	100%	93%	60%	12%	12%
	Pectoral fin	100%	70%	53%	2%	5%
	Operculum	–	–	7%	–	–
	Buccal cavity	–	–	5%	–	–
	Head	–	–	–	–	–
	Lateral line	–	–	–	–	–
Cataract		–	–	–	–	–
Exophthalmus		–	–	2%	–	–

Clinical signs of GBD are given as prevalence of observations (%). The treatment groups (T) were exposed to TDG (%) corresponding to the number in their name. No clinical signs detected (0%) are marked as negative (-).

Abnormal swimming behaviour was noted for fish exposed to TDG 115% and 120%. Some fish showed erratic swimming patterns, while others were apathetic and did not respond to any stimuli. This behaviour was noted for fish in T120 after only 90 minutes of exposure to DGS, while fish from T115 showed more erratic behaviour at start of the trial, followed by apathy and increased moribundity and mortality within 24 hours of exposure. Moribund fish swam at the surface. A few fish from group T110 also became apathetic (no response to stimuli) after 8 days of exposure. Gas bubbles released through mouth (burping) were observed in all groups including control group (T100), but frequency appeared to be higher among fish from T110, T115, and T120. Fish in groups exposed to TDG < 115% stayed close to the bottom of the tanks and displayed calm behaviour with low swimming activity when not disturbed.

#### Atlantic salmon alevins (trial 2a).

**Fig 10:**
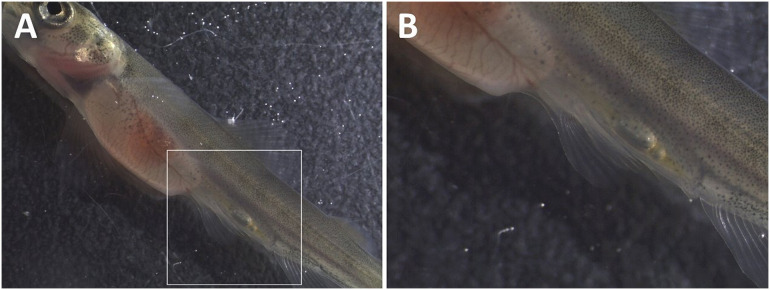
Clinical signs of gas bubble disease in alevin. Appearance of gas bubbles in the intestine to Atlantic salmon (*Salmo salar*) alevin exposed to 120% total dissolved gas supersaturation for 12 days. Photo (B) is an enlarged part of photo (A).

No mortalities or subcutaneous emphysema were observed among alevins in the groups during the 14 days of exposure. The only signs that could be related to gas supersaturation among alevins were gas bubbles in the body cavity (gastrointestinal tract; Fig 10). These signs were found in fish from groups T120 (5 of 30 fish) and T115 (1 of 30 fish) and appeared after 12 days of exposure.

**Fig 11:**
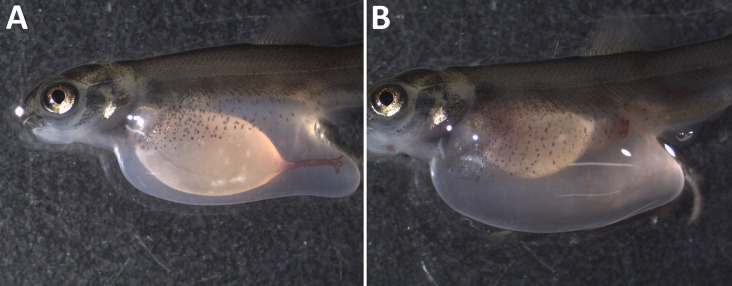
Blue sac disease in Atlantic salmon (*Salmo salar*) alevin. Ascites fluid in yolk sac to alevins that have been exposed to (A) 110% and (B) 115% total dissolved gas supersaturation for two weeks.

Gas bubbles in the gastrointestinal tract were not detected in any fish after one to three days of recovery. However, morphological abnormalities, such as a swollen yolk sac due to excessive fluid (yolk sac edema), occurred among two alevins from each of T110 and T115 after 4 days of recovery (last sampling). These abnormalities were diagnosed as blue sac disease (Fig 11).

No abnormal swimming behaviour among alevins in any group were noted during the treatment period.

#### Atlantic salmon fry (trial 2b).

**Fig 12:**
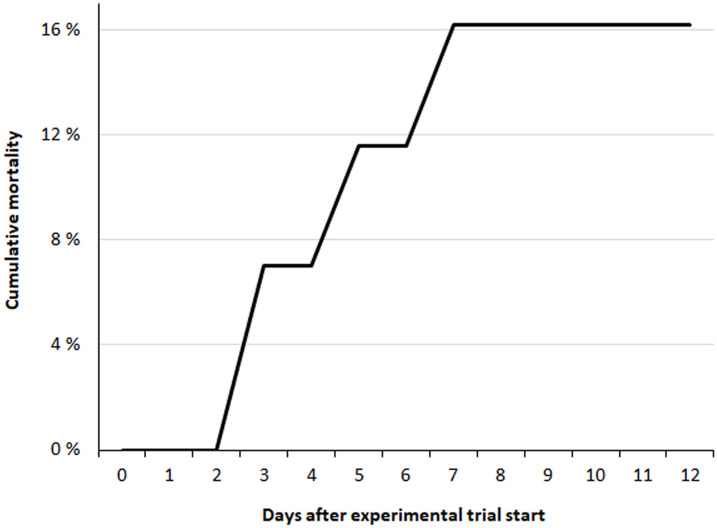
Lethal TDG supersaturation levels for salmon fry. Cumulative mortality of Atlantic salmon (*Salmo salar*) fry exposed to 120% total dissolved gas (TDG) supersaturation (n = 43).

**Fig 13:**
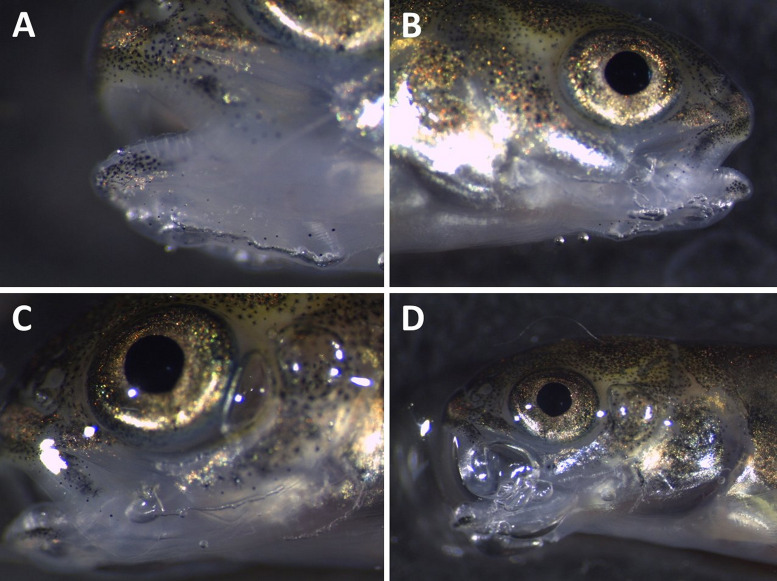
Clinical signs of subacute gas bubble disease in Atlantic salmon (*Salmo salar*) fry. Gas bubble formation (subcutaneous emphysema) in the lower jaw **(A, B)**, in lower jaw, operculum, and behind the eye **(C)**, in jaws, buccal cavity, and operculum **(D)**.

Mortalities among fry occurred in group T120 after three days of exposure (Fig 12). All dead and moribund fish exhibited external signs of GBD, such as subcutaneous emphysema in the head, operculum, or in the buccal cavity (Fig 13).

**Fig 14:**
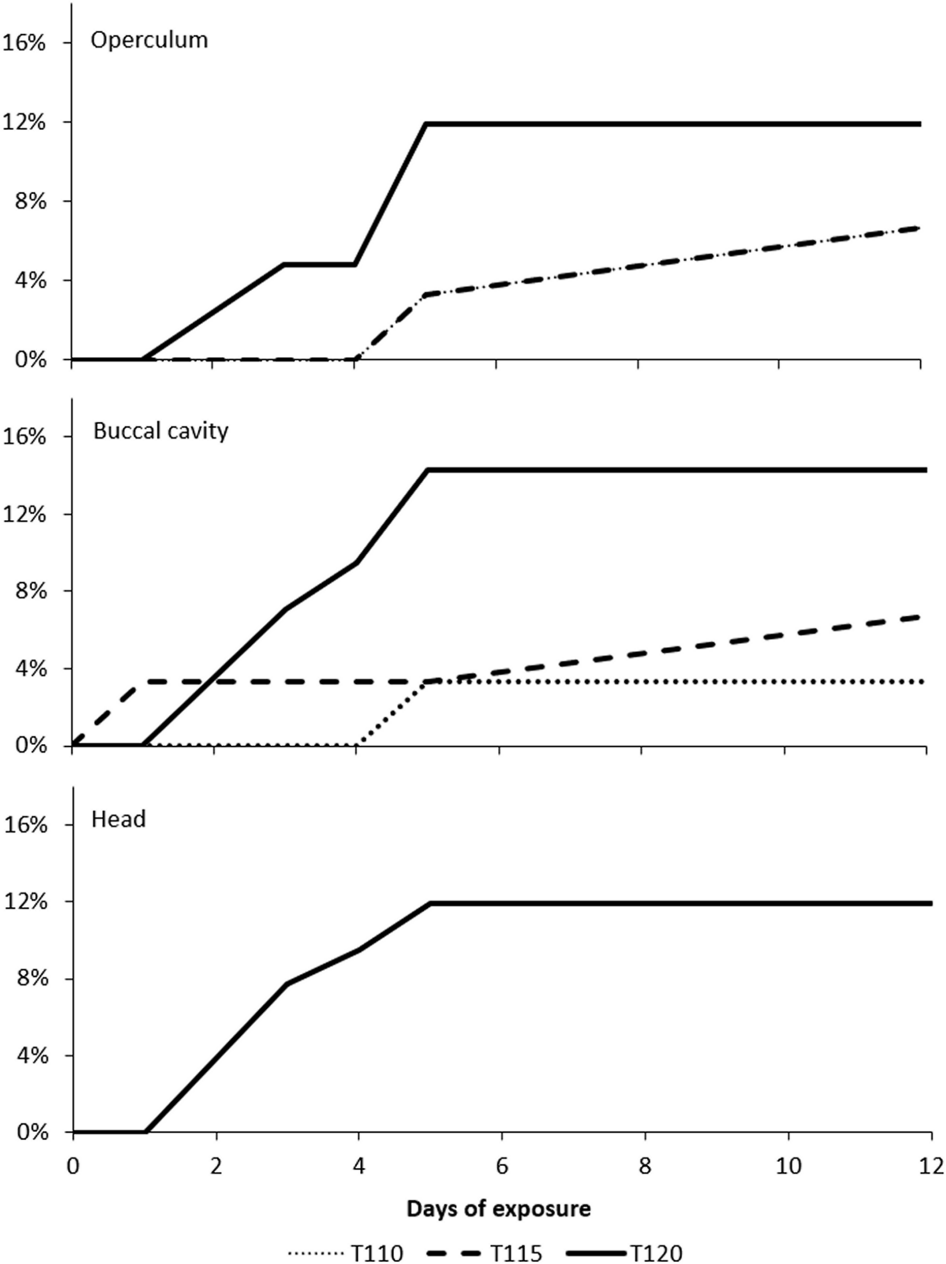
Cumulative frequencies of observed subcutaneous emphysema in Atlantic salmon (*Salmo salar*) fry. Occurrence of gas bubbles in operculum, buccal cavity, and head region of fry exposed to 110% (n = 30), 115% (n = 30) and 120% (n = 30) total dissolved gas. Gas bubbles on the head region were only observed in fry exposed to 120% total dissolved gas (TDG) supersaturation.

There was a significant increase in the incidence of emphysema among moribund or dead fish compared to live fish in T120 (FET; p < 0.001). Subcutaneous emphysema in the operculum and buccal cavity were also observed in a few fish in groups T115 and T110 after one and five days of exposure, respectively. The frequency of emphysema among fish from groups T110 and T115 was not significantly different from fish in T100 (control) (FET; p > 0.05). Progress in formations of gas bubbles is shown in Fig 14. No emphysema or other gross pathology associated with GBD were detected among fry in the groups T105 and T100.

**Fig 15:**
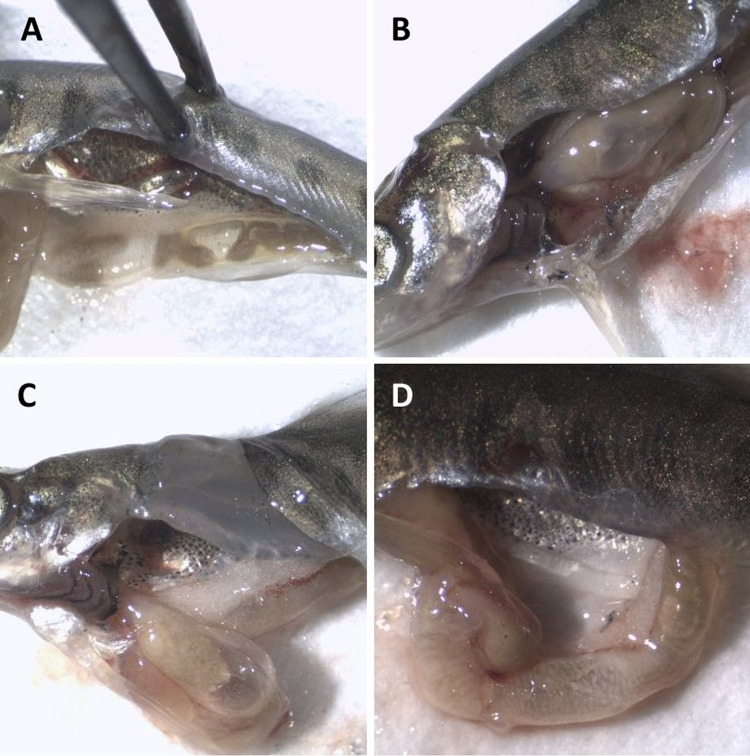
Observations of as bubbles in the gastrointestinal (GI) tract of salmon fry. Observed gas bubbles in the GI tract of Atlantic salmon (*Salmo salar*) fry exposed to total dissolved gas (TDG) supersaturation. **(A)** Gas in the intestine after exposure of 120% TDG for 5 days; **(B)** Gas bubbles in stomach and intestine after exposure of 110% TDG for 12 days; **(C)** Gas in the stomach after exposure of 120% TDG for 12 days; **(D)** Gas bubbles in the intestine after exposure of 115% TDG for 5 days.

**Fig 16:**
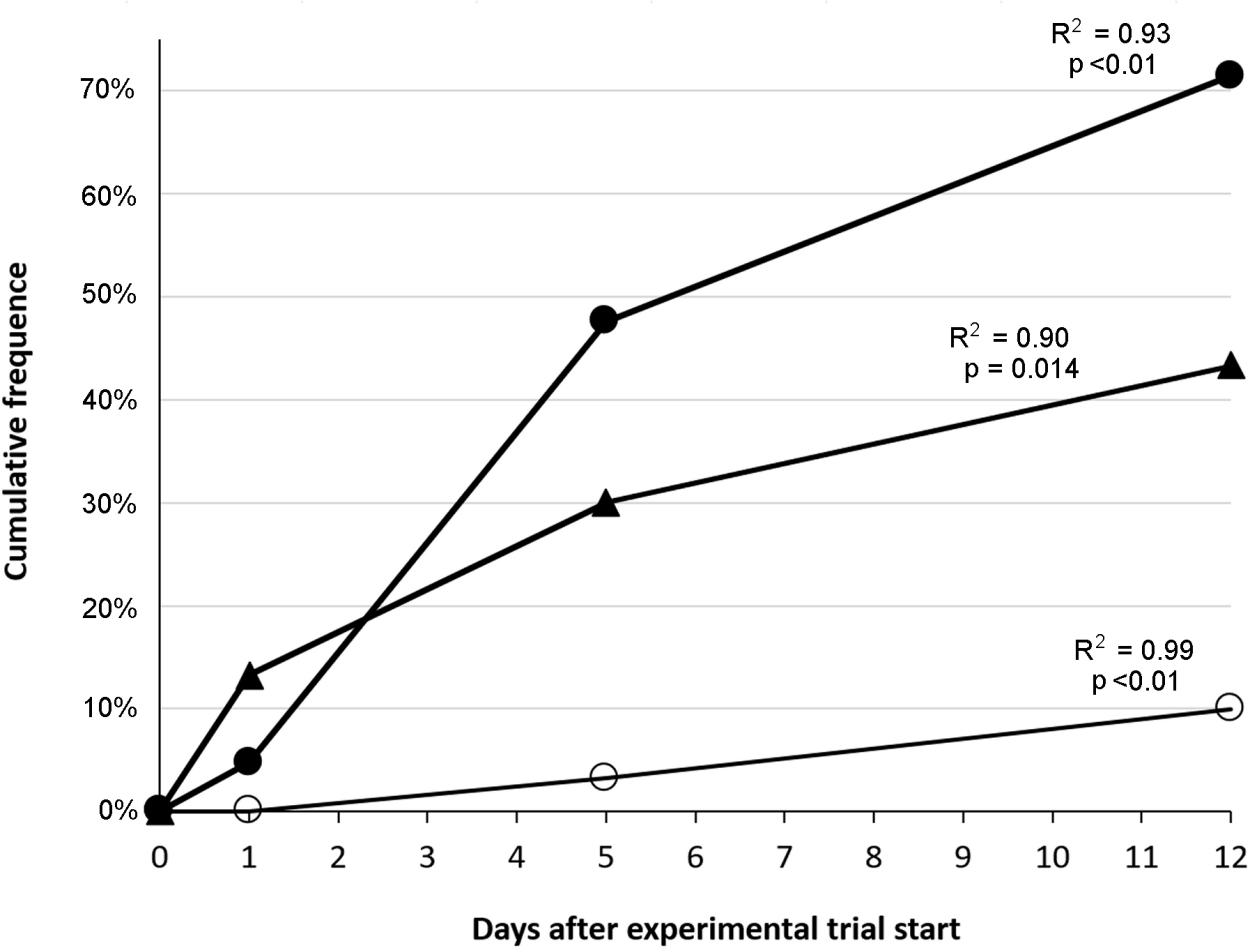
Rates of gas bubble formations in the gastrointestinal (GI) tract of salmon fry. Cumulative frequency (%) of observed gas bubbles in the GI tract of Atlantic salmon (*Salmo salar*) fry exposed to total dissolved gas supersaturation of 110% (T110; ○), 115% (T115; ▲), and 120% (T120; ●). Each dot represents 10 fish. The frequency expressed as a linear regression (fitted line) for T110 (y = 0.0086x − 0.0055), T115 (y = 0.0329x + 0.0683), and T120 (y = 0.061x + 0.0352). The respective coefficients of determination (R^2^) and p-values are shown in the figure.

Other signs that appeared among Atlantic salmon fry were large and easily detectable gas bubbles in the gastrointestinal tract (Fig 15). This was most prominent among fish in groups T110, T115, and T120, and there was also a tendency towards increasing gas bubble formation throughout the experimental period in these groups (Fig 16). Linear regression analyses show that rates of gas bubble formations in the gastrointestinal tract to fish in these groups were significant different (p < 0.05) compared to fish in T100 (control group), which had no observable formation of bubbles in the gastrointestinal tract.

Gas bubbles in the gastrointestinal tract could still be detected among fry in groups T110 (2 of 10 fish) and T115 (3 of 10 fish) after a day of recovery at 100% TDG, while no bubbles were detected in these groups in the last sampling after four days of recovery. Gas bubbles in the intestine were only found in one fish from group T120 in this last sampling. Hence, recovery caused a significant reduction in the frequency of bubbles in the gastrointestinal tract among fish from T120 (FET, p = 0.01). No other gas bubbles or signs associated with GBD could be observed among fish in the groups at the end of the recovery period. The prevalence of observed clinical signs associated with GBD in trial 2 is summarised in Table 5.

**Table 5:** Clinical signs of gas bubble disease (GBD) in Atlantic salmon fry (*Salmo salar*) from trial 2b.

Groups		T120	T120†	T115	T115†	T110	T110†	T105	T105†	T100
Exposure time (days)		13	4	13	4	13	4	13	4	17
No. fish examined		43	7	30	20	30	20	30	20	50
Mortality		16%	–	–	–	–	–	–	–	–
Gill emboli		–	–	–	–	–	–	–	–	–
Hemorrhagic ascites		–	–	–	–	–	–	–	–	–
Emphysema	Caudal fin	–	–	–	–	–	–	–	–	–
	Dorsal fin	–	–	–	–	–	–	–	–	–
	Anal fin	–	–	–	–	–	–	–	–	–
	Pelvic fin	–	–	–	–	–	–	–	–	–
	Pectoral fin	–	–	–	–	–	–	–	–	–
	Operculum	12%	–	7%	–	7%	–	–	–	–
	Buccal cavity	14%	–	7%	–	3%	–	–	–	–
	Head	12%	–	–	–	–	–	–	–	–
	Lateral line	–	–	–	–	–	–	–	–	–
Bubbles in the GI tract		71%	14%	43%	15%	10%	10%	–	–	–
Cataract		–	–	–	–	–	–	–	–	–
Exophthalmus		–	–	–	–	–	–	–	–	–

Clinical signs of GBD presented as total prevalence of observations (%). The groups (T) were exposed to TDG levels (%) corresponding to the numbers in their names. The trial included a four-day recovery period where TDG levels were set to 100% in all treatment groups (†). GI: Gastrointestinal. No clinical signs detected (0%) are marked as negative (-). Mortality includes moribund fish.

Abnormal swimming behaviour was noted in fry exposed to TDG 120%. A few fish showed erratic swimming patterns, and some swam close to the surface, clearly struggling with positive buoyancy. Most fish in all groups remained near the bottom and displayed low swimming activity.

#### Brown trout parr (trial 3).

**Fig 17:**
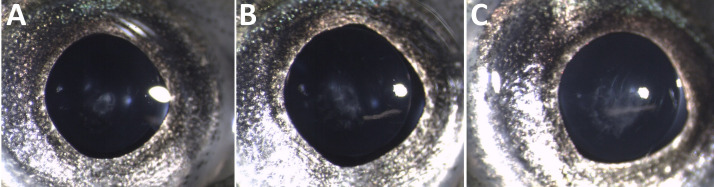
Eye damage. Cataract in brown trout (*Salmo trutta*) parr exposed to 115% total dissolved gas supersaturation for 48 hours. **(A)** Cataract covering 6% of the lens, **(B)** 12% of the lens; **(C)** 23% of the lens.

The acute phase of GBD, fish from groups T120 and T115 exhibited prominent clinical signs, including gas emboli in the gills, subcutaneous emphysema in the fins, and bubbles along the lateral line. A few fish in T115 and T120 also developed bubble formation in the buccal cavity, detected at 48 hours and 11 hours after exposure, respectively. Furthermore, cataracts were identified in six of 33 fish from T115 (Fig 17). No cataracts were detected in fish from the remaining groups in this trial.

**Fig 18:**
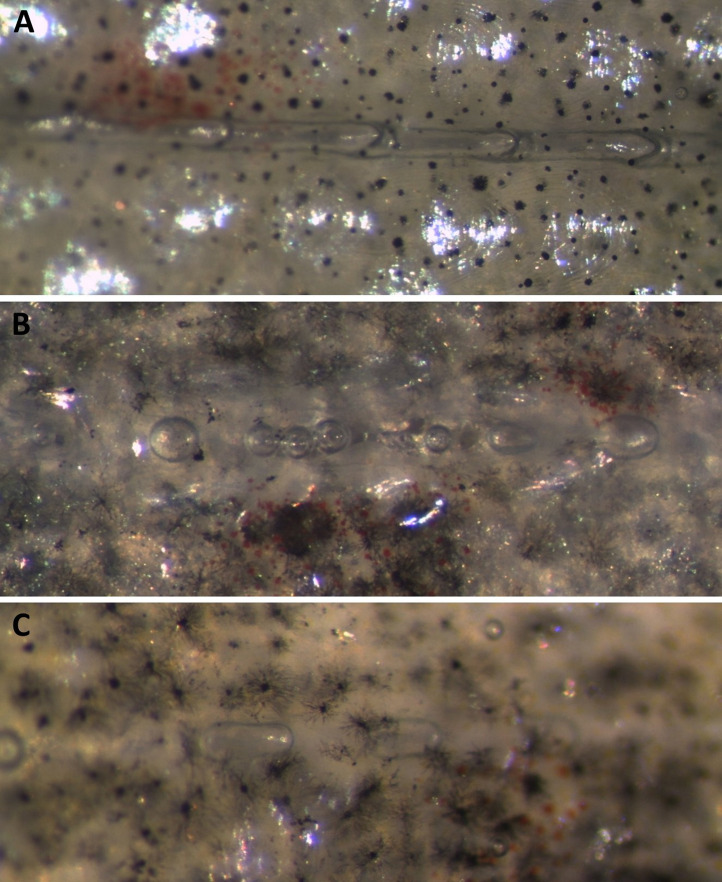
Lateral line. Gas bubble formation in the lateral line of brown trout (*Salmo trutta*) parr exposed to total dissolved gas supersaturation of 120% **(A, B)** and 100% **(C)**. **(A)** Total occlusion of the lateral line by gas bubbles; **(B)** Scattered bubble formation; **(C)** Scattered bubble formation.

The most common sign of GBD among brown trout parr was the presence of gas bubbles in the lateral line, occurring across all groups, including the control group (T100) and group T105. Appearance of gas bubbles in the lateral line is shown in Fig 18.

**Fig 19:**
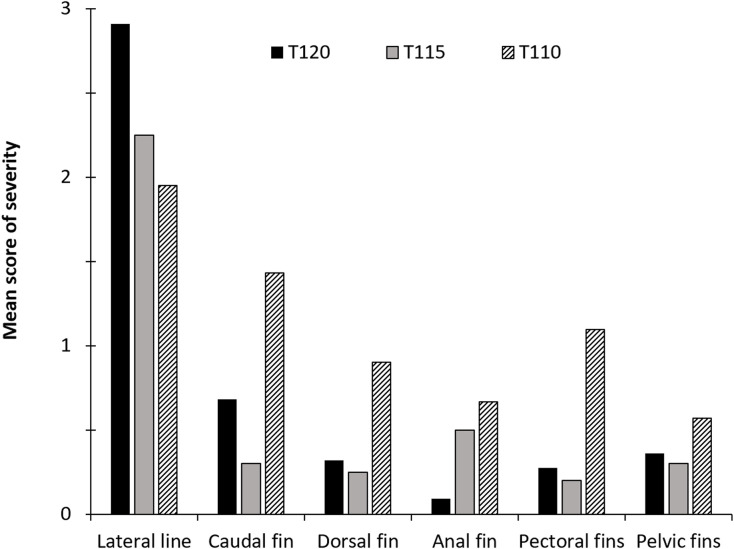
Ranked severity of gas bubble formation in fins and lateral line of brown trout exposed to different levels of TDG supersaturation. The mean intensity of gas bubbles scored on a scale that indicate the coverage of bubbles on the fins or lateral line of brown trout (*Salmo trutta*) parr: 0 = No bubbles observed, 1 = less than 25% covered with bubbles, 2 = 26-50%, and 3 = more than 50%. The treatment groups (T) were exposed to total dissolved gas (TDG) supersaturation (%) according to the number in their name. T120 (n = 22) was exposed for 13 hours, T115 (n = 20) was exposed for 48 hours, and T110 (n = 21) was exposed for 14 days.

A few small bubbles, extending less than 10% of the length of the lateral line, were easily detected in a total of 7 out of 50 examined fish from both T100 (control group) and T105 using a dissecting microscope (Table 6 and Fig 18). The bubbles in the lateral line of fish from T100 and T105 were observed after 14 days of exposure (last sampling before recovery). Insignificant difference in the frequency of observed gas bubbles in the lateral line were observed between fish in T105 and T100 (FET; p < 0.05). However, a statistically significant difference in the intensity gas bubbles in the lateral line was observed among fish from T110, T115, and T120 compared to T100 (control), as indicated by the Kruskal-Wallis; p < 0.0001). The highest frequency of bubbles in the lateral line was observed in the acute phase of GBD among fish from both groups T120 and T115 (FET; p < 0.04), occurring with a prevalence ranging from 95% to 100% and an average severity score higher than 2. Insignificant difference in the observed frequency of this sign were found between moribund or dead fish and euthanized fish. The average score of ranked severity for bubble formation in the lateral line was significantly higher in fish from group T120 within 13 hours of exposure compared to fish from T110 after 14 days of exposure to DGS (Mann–Whitney U, p = 0.007). There was an insignificant difference in fish from T115 after 48 hours of exposure compared to T110. The mean score of severity in the lateral line and fins for fish from the treatment groups is summarised in Fig 19.

**Table 6:** Clinical signs of gas bubble disease (GBD) in bown trout parr (*Salmo trutta*) from trial 3.

Treatment groups		T120	T115	T110	T110†	T105	T105†	T100
Exposure time		12 hrs	48 hrs	14 days	7 days	14 days	7 days	21 days
No. fish examined		22	20	79	16	70	20	90
Mortality		50%	50%	16%	–	–	–	–
Gas emboli gills		73%	35%	35%	–	–	–	–
Hemorrhagic ascites		–	–	6%	–	–	–	–
Emphysema	Caudal fin	64%	25%	35%	–	–	–	–
	Dorsal fin	23%	25%	14%	–	–	–	–
	Anal fin	9%	20%	20%	–	–	–	–
	Pelvic fin	27%	20%	18%	–	–	–	–
	Pectoral fin	27%	20%	30%	–	–	–	–
	Operculum	–	–	35%	–	–	–	–
	Buccal cavity	14%	10%	18%	–	–	–	–
	Lateral line	100%	95%	53%	–	14%	–	14%
Cateract		–	15%	–	–	–	–	–
Exophthalmus		–	–	9%	6%	–	–	–

Clinical signs of GBD presented as total prevalence of observations (%). The treatment groups (T) were exposed to TDG levels (%) corresponding to the numbers in their names. The trial was followed by a one-week recovery trial where TDG was set to 100% for all treatment groups (†). Mortality includes moribund fish. No clinical signs detected (0%) are marked as negative (-).

**Fig 20:**
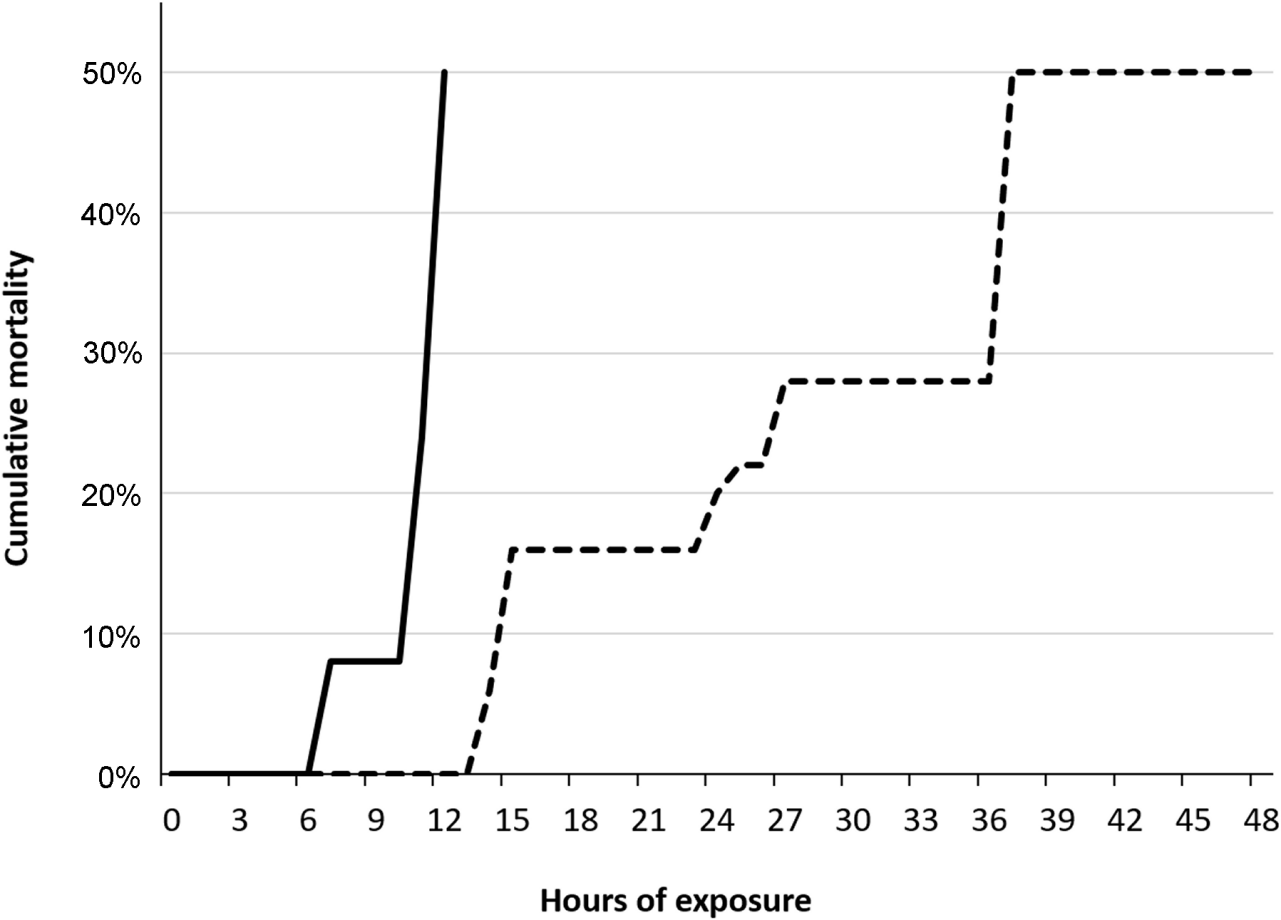
Lethal TDG supersaturation levels for brown trout during acute gas bubble disease. Cumulative mortality of brown trout (*Salmo trutta*) parr exposed to 115% (dotted line, n = 50) and 120% (solid line, n = 50) total dissolved gas (TDG) supersaturation.

Emphysema in fins was observed in brown trout parr from the groups T120, T115, and T110, with a significantly higher frequency than in T100 (control; p < 0.05). The highest prevalence was observed in the caudal fin from T120 (64%). The ranked severity of gas bubble formation in the caudal fin was significantly higher (Kruskal-Wallis; p < 0.02) in T110 after 14 days of exposure and in T120 after 13 hours of exposure compared to T100 (control; 14 days of exposure). Overall, there were insignificant difference in the prevalence or ranked severity of observed emphysema in unpaired or paired fins in fish that developed acute GBD (T120 and T115) within 48 hours compared to fish that were exposed to TDG 110% for 14 days (T110). Additionally, there were insignificant difference in the appearance of these signs when moribund or dead fish were compared with euthanized fish (FET; p > 0.05). T120 and T115 were terminated within two days post-exposure to DGS due to acute GBD and high mortality. Mortality is associated with gas emboli in gills, and these signs were significantly more frequent among moribund or dead fish compared to euthanized fish in both T120 and T115 (FET; p < 0.003). A cumulative mortality of 50% (LT_50_) occurred within 12 hours and 37 hours among fish in T120 and T115, respectively (Fig 20).

**Fig 21:**
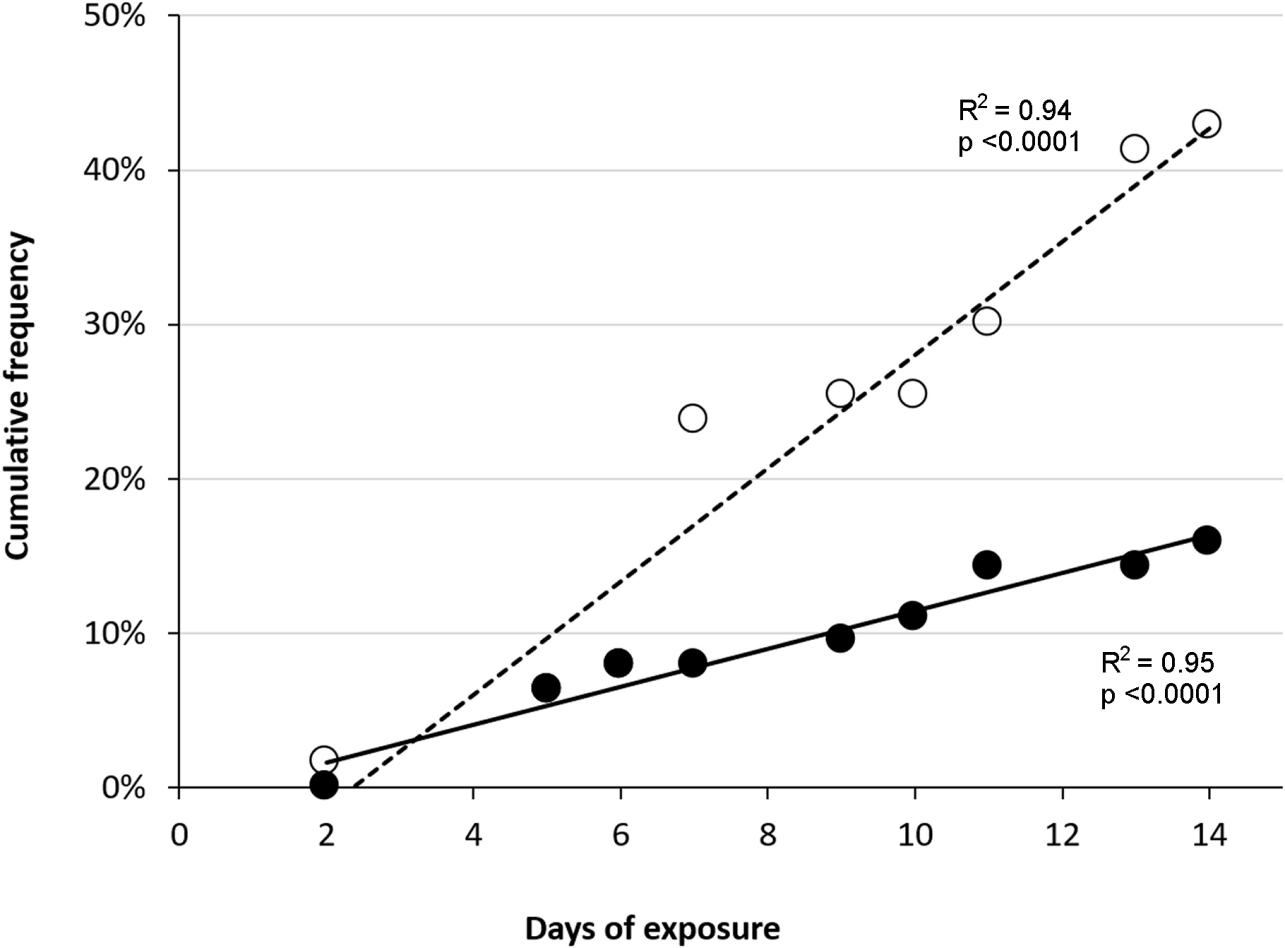
Gill embolism and mortality rates observed in brown trout during subacute gas bubble disease. Cumulative frequency of observed gill embolism and mortality or moribund brown trout (*Salmo trutta*) parr in group T110 exposed to 110% total dissolved gas supersaturation (TDG) over 14 days. Cumulative frequency of observations is expressed as a linear regression (fitted line) for gas embolism in gills (○; y = 0.0367x − 0.0867) and for mortality or moribund fish (●; y = 0.0123x − 0.0079). The respective coefficients of determination (R^2^) and p-values are shown in the figure.

In group T110, gas embolism in gills occurred subacutely among brown trout after one day of exposure, with the prevalence of gill emboli increasing during exposure time, showing a cumulative bubble formation rate of 3.7% per day (Fig 21). Moribund fish occurred after four days of exposure, with a cumulative mortality rate of 1.2% per day over two weeks (Fig 21). Gas emboli occurred more frequently among moribund or dead fish in T110 compared to euthanized fish (FET; p = 0.0015).

**Fig 22:**
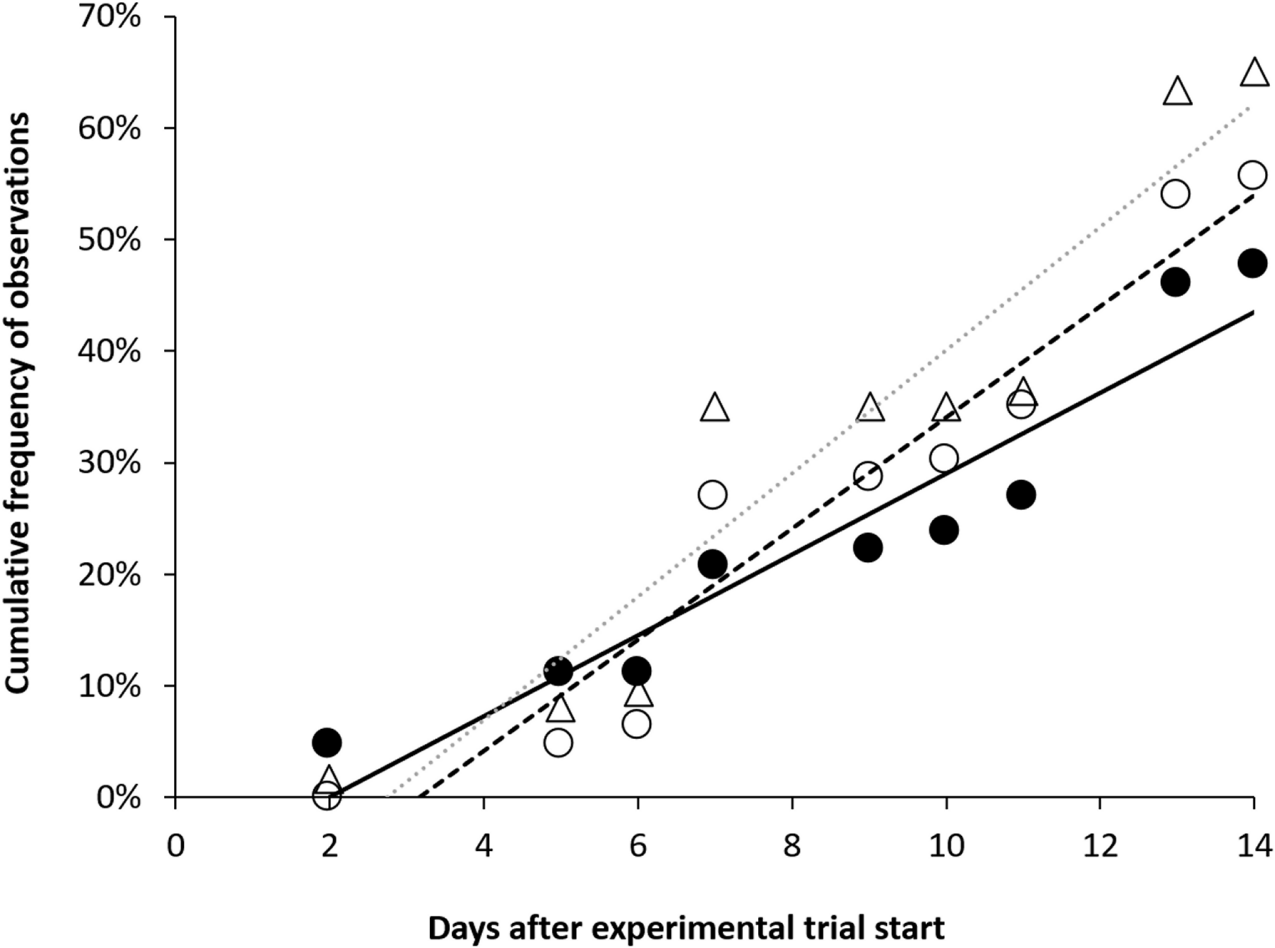
Rates of gas bubble formation in the fins and lateral lines of brown trout during subacute gas bubble disease. Progression of bubble formation in brown trout (*Salmo trutta*) parr exposed to 110% total dissolved gas supersaturation. Cumulative prevalence of bubble formation in unpaired fins (○; caudal and anal), paired fins (●; pectoral and pelvic) and lateral line (△). Formation of bubbles expressed as linear regression for unpaired fins: y = 0.0498x-0.1581; paired fins: y = 0.0362x-0.0719; lateral line: y = 0.0552x-0.1511. R^2^ (coefficient of determination) > 0.89 for all.

Hemorrhagic ascites was observed in five trout parr from T110 after seven to nine days of exposure, but this symptom was only observed among moribund or dead fish. There were insignificant differences in the progression of gas bubble formation in unpaired fins, paired fins, and along the lateral line in T110 during 14 days of exposure when comparing slopes in a linear regression (p < 0.05). The prevalence of these symptoms increased by 3% to 6% per day (Fig 22). The severity of bubble formation also increased over time, and this increase was statistically significant for unpaired fins (Kendall’s τ = 0.27, p < 0.002), paired fins (Kendall’s τ = 0.23, p < 0.007), and lateral line (Kendall’s τ = 0.39, p < 0.0001).

Abnormal swimming behaviour was noted for fish exposed to TDG 120%. The fish showed erratic swimming patterns followed by apathetic behaviour within 3 hours. There were no signs of disturbed buoyancy, and all fish stayed near the bottom of the tank. A few fish in group T115 (TDG 115%) showed erratic swimming patterns close to the surface. In general, the fish from T115 showed higher response to stimuli than fish from T120. No abnormal behaviour was noted for fish in the other groups, including the control group (T100) that spent most of their time near the bottom and displayed calm behaviour when undisturbed.

**Fig 23:**
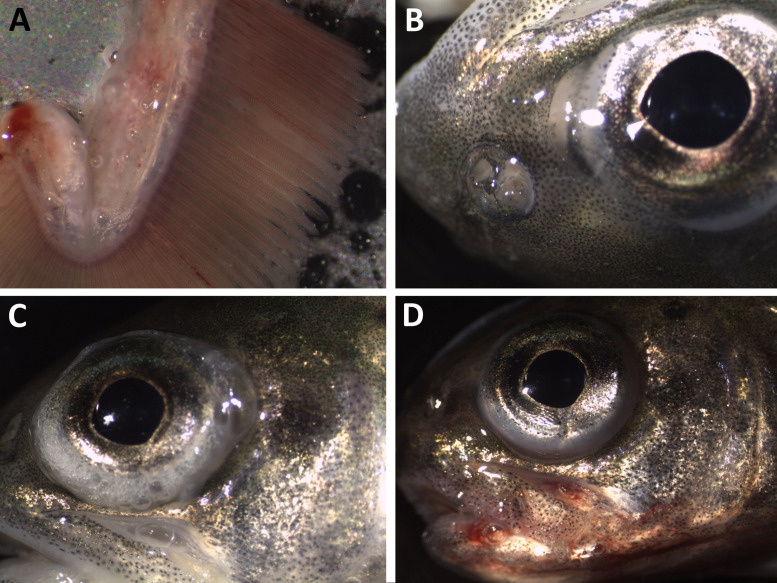
Clinical signs of gas bubble disease in trout during subacute gas bubble disease in brown trout. Subacutes signs of gas bubble disease in brown trout (*Salmo trutta*) parr exposed to 110% total dissolved gas supersaturation. **(A)** Bubble formation on the gill arches; **(B)** Gas bubbles in the nostrils; **(C)** gas bubble formation in the choroid gland in the eye of a specimen exhibiting exophthalmia; **(D)** Hemorrhages in the head region.

**Fig 24:**
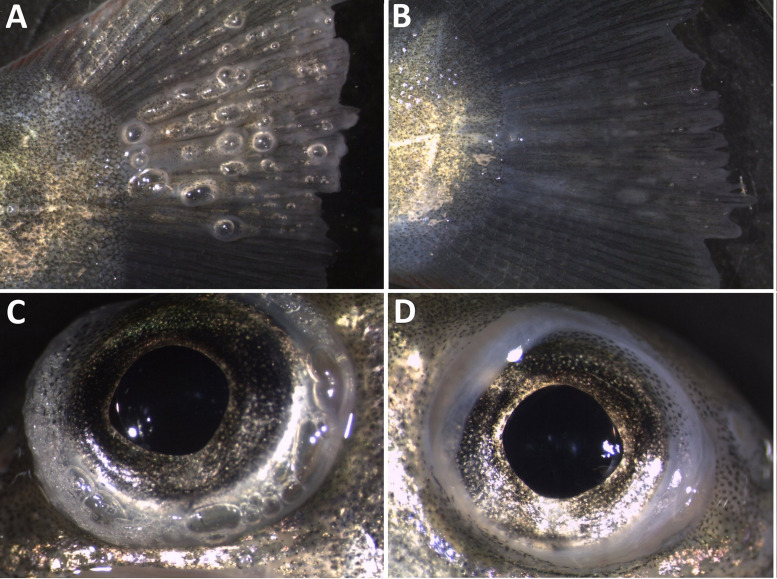
Recovery from gas bubble disease in brown trout (*Salmo trutta*) parr. **(A)** Subcutaneous emphysema (gas bubbles) in the caudal fin after exposure to 110% total dissolved gas supersaturation (TDGS) for 13 days; **(B)** Scars in caudal fin after seven days of recovery in 100% TDG; **(C)** Gas bubbles in the choroid gland in the eye after 110% TDGS for 13 days; **(D)** Choroid gland after seven days of recovery in 100% total dissolved gas.

Most frequently observed GBD signs of brown trout parr from trial 3 are summarised in Table 6. Less frequent signs, such as bubble formation on the gill arches, in the nostrils, in the choroid gland of the eye, and haemorrhages in the head region, were only observed in brown trout exposed to 110% TDG (T110) for more than 13 days (Fig 23). In T110, 75% of the brown trout parr developed subcutaneous emphysema in the fins and lateral line (Figs 18 and 24A). These symptoms were not observed in any fish after seven days of recovery. Scars, apparently originating from emphysema, were clearly visible as pale marks in the caudal fins of 9 out of 16 fish examined (Fig 24B). There were no statistically significant differences in the frequency of exophthalmia among fish in T110 after seven days of recovery in 100% TDG (FET, p > 0,05). However, the choroid gland of the eye was marked and opaque, yet without visible gas bubbles in some fish after seven days of recovery, indicating some degree of recovery (see Figs 24C and 24D).

#### Interspecific variation among salmonid parr (trial 1 and 3).

Observed GBD signs of Atlantic salmon parr exposed to TDG 120% in trial 3 was similar to those of Atlantic salmon from group T120 in trial 1, albeit with a lower frequency and intensity of observed emphysema in fins (FET, p < 0.001). Overall, the frequency and severity of fin emphysema were significantly higher in Atlantic salmon from trial 1 than in salmonid parr from trial 3, including brown trout (FET, p < 0.001). There were no statistically significant differences in ranked severity of emphysema in fins among Atlantic salmon, brown trout, or rainbow trout from trial 3.

Gas bubbles in the lateral line were only observed in brown trout and rainbow trout from trial 3, with 100% observation prevalence in fish exposed to TDG 120% over the duration until 50% mortality or moribundity occurred (LT_50_). The Intensity of gas bubble formations in the lateral line was most pronounced among brown trout (FET, p = 0.016).

Hemorrhagic ascites occurred in rainbow trout with the same frequency as in Atlantic salmon from Trial 1 exposed to TDG 120% (FET, p = 1). Also, the frequency of these signs in rainbow trout were higher among dead or moribund fish compared to euthanized fish (FET, p = 0.007). Hemorrhagic ascites was not observed in the examined brown trout and were noted only in a few Atlantic salmon parr from trial 3 after three hours of exposure to 120% TDG.

**Fig 25:**
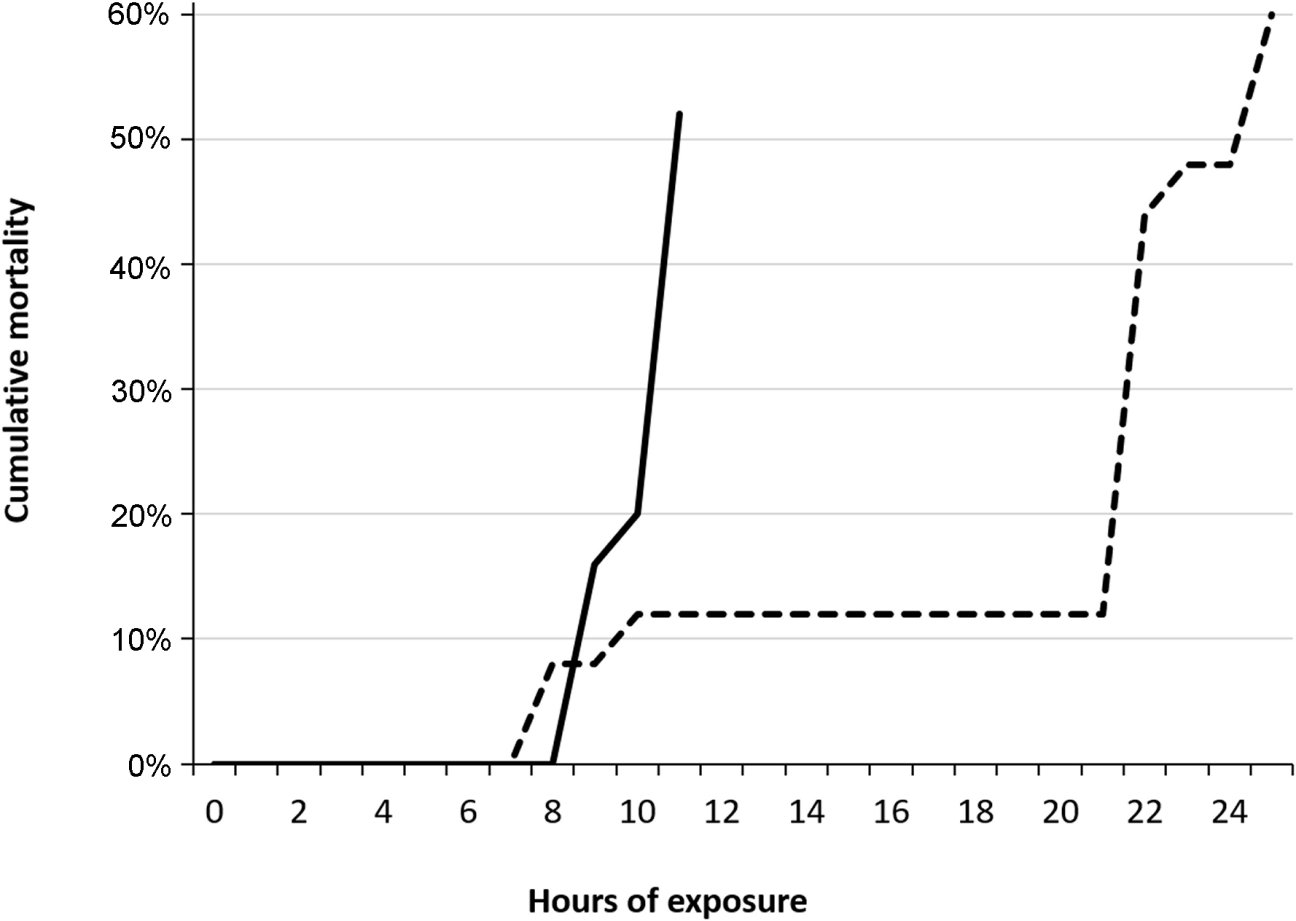
Lethal TDG supersaturation levels for salmon and rainbow trout parr during acute gas bubble disease in Trial 3. Cumulative mortality in parr of Atlantic salmon (*Salmo salar*, solid line; n = 16) and rainbow trout (*Oncorhynchus mykiss*, dotted line; n = 16) exposed to 120% total dissolved gas supersaturation.

The frequency of gas emboli in the gills of salmonid parr from group T120 in trial 3 was significantly higher among dead or moribund fish compared to euthanized fish (FET, p < 0.003). No statistically significant differences were found in the frequency or severity of gill emboli between salmonid parr from group T120 in trials 1 and 3, although the mortality rate was higher in Atlantic salmon parr in trial 1 compared to salmonids in trial 3 (Figs 5, 20 and 25).

Atlantic salmon from trial 1 exhibited a significantly higher mortality rate than salmonid parr from the other trial groups (FET, p < 0.00001), reaching LT_50_ after only 4 hours of exposure to 120% TDG.

Brown trout and Atlantic salmon from trial 3 reached LT_50_ after 12 hours and 15 hours exposure to 120% TDG, respectively. Overall, the mortality rate within 15 hours of exposure to 120% TDG was significantly higher among Atlantic salmon and brown trout compared to rainbow trout (FET, p < 0.003). Rainbow trout parr exhibited the lowest mortality rate, reaching LT_50_ after 25 hours of exposure. Average mortality rates, calculated as the number of dead or moribund fish per hour with the duration at LT_50_, yielded a ranked mortality rate of 7.5 for Atlantic salmon in trial 1, 2.1 for brown trout in trial 3, 1.2 for salmon in Trial 3, and 0.5 for rainbow trout in trial 3.

Atlantic salmon and brown trout parr started to show apathetic behaviour within 3 hours of exposure to 120% TDG. In general, Atlantic salmon and brown trout parr showed less flight response to external stimuli than rainbow trout parr when exposed to 120% TDG. Gas bubbles released from the mouth (‘burping’) were observed among Atlantic salmon parr, but not among brown trout or rainbow trout.

The observed GBD signs of Atlantic salmon parr and rainbow trout parr from trial 3 are summarised in Table 7.

**Table 7:** Clinical signs of gas bubble disease (GBD) in Atlantic salmon parr (Salmo salar) and rainbow trout (Oncorhynchus mykiss) from trial 3.

Species		Salmon	Rainbow trout
Exposure time		15 hrs	25 hrs
Total n		25	25
No. fish examined		16	16
Mortality		52%	52%
Gas emboli gills		81%	81%
Hemorrhagic ascites		19%	75%
Emphysema	Caudal fin	13%	19%
	Dorsal fin	13%	–
	Anal fin	19%	–
	Pelvic fin	31%	13%
	Pectoral fin	25%	44%
	Operculum	–	6%
	Buccal cavity	19%	–
	Head	–	–
	Lateral line	–	100%
Cataract		6%	–
Exophthalmus		–	–

Clinical signs of GBD among Atlantic salmon parr and rainbow trout parr exposed to 120% TDG. The signs are given as total prevalence of observations (%). No clinical signs detected (0%) are marked as negative (-). Mortality includes moribund fish.

#### European minnow (trial 4).

**Fig 26:**
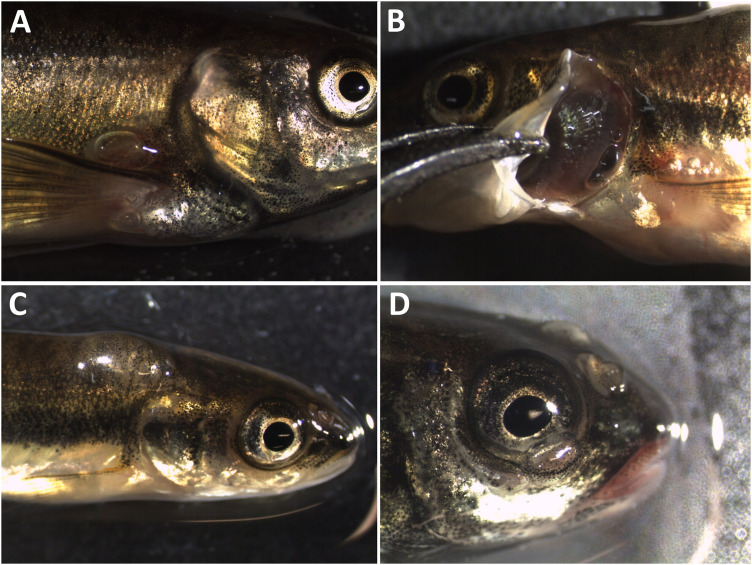
Clinical signs of gas bubble disease in adult European minnow (*Phoxinus phoxinus*) exposed to 120% total dissolved gas supersaturation. A: Subcutaneous emphysema (gas bubble) at the basis of the pectoral fin; B: Gas bubble formation in the gill chamber and basis of the pectoral fin; C: Gas bubble formation on the neck; D: Gas bubble formation on the head region, including operculum, nostrils, and eye.

Subcutaneous emphysema in fins of European minnow was rare and only detected in the pectoral fin bases of two fish from group T120 after more than four days of exposure. Other signs associated with GBD included gas bubbles in the head region, the operculum, and in the gill chamber (Fig 26). Gas emboli in gills were only detected in one dead fish from T120 after more than 10 days of exposure. The frequency of observed signs was not significantly different from that of fish in the control group (T100; FET > 0.1).

**Fig 27:**
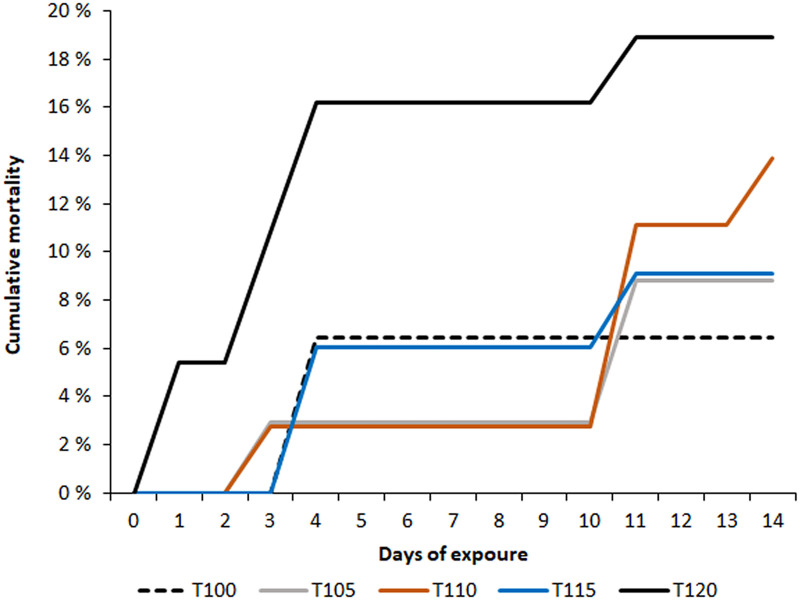
Lethal TDG supersaturation levels for adult minnow. Cumulative mortality of adult European minnow (*Phoxinus phoxinus*). The treatment groups (T) were exposed to total dissolved gas (TDG) supersaturation (%) according to the number in their name.

A cataract in the eye of one fish from group T110 was observed after 10 days of exposure. This cataract showed low opacity and covered less than 50% of the lens. No other signs or clinical signs of GBD were detected in this individual. The mortality rate was highest in group T120 (Fig 27), but most of the dead or moribund fish in all groups did not show any signs or gross pathology that could be associated with GBD. The cause of death remained unknown in these cases.

Fish from groups T115 and T120 showed less response to stimuli compared to fish from the other groups, exhibiting a reduced flight response when collected for examination. Fish in all groups mostly remained at the bottom and exhibited low swimming activity. Clinical signs of GBD in European minnow are shown in Figs 26 and 27 and summarised in Table 8.

**Table 8:** Clinical signs of gas bubble disease (GBD) in adult European minnow (*Phoxinus phoxinus*) from trial 4.

Groups		T120	T115	T110	T105	T100
Exposure time		14 days	14 days	14 days	14 days	14 days
No. fish examined		36	33	36	33	30
Mortality		19%	9%	14%	9%	6%
Gas emboli gills		3%	–	–	–	–
Hemorrhagic ascites		–	–	–	–	–
Emphysema	Caudal fin	–	–	–	–	–
	Dorsal fin	–	–	–	–	–
	Anal fin	–	–	–	–	–
	Pelvic fin	–	–	–	–	–
	Pectoral fin	6%^a^	–	–	–	–
	Operculum	6%	–	–	–	–
	Gill chamber	11%	–	–	–	–
	Head	8%	3%	3%	–	–
	Lateral line	–	–		–	–
Cataract		–	–	3%	–	–
Exophthalmus		–	–	–	–	–

The clinical signs of GBD are given as total prevalence of observations (%). Mortality includes moribund fish. No symptoms detected (0%) are marked as negative (-).

^a^Basis of pectoral fins.

## Discussion

The experimental trials showed that tolerance of fish to TDGS differed among species and among life stages. Acute GBD only occurred among salmonid parr (Atlantic salmon, brown trout, and rainbow trout) from trial 1 and 3 that were exposed to TDG ≥ 115%. Most of the common clinical signs of acute GBD were manifested, including gas embolism in gills with a high prevalence (>70%) when exposed to 120% TDG, and this sign highly correlates with mortality or moribundity, consistent with other research [4,8,33,34]. However, there were both inter- and intraspecific differences in mortality rates among parr exposed to similar levels of TDGS. Published inter- and intraspecific tolerances to TDGS in salmonid parr are summarised in Table 9, showing that the tolerance measured as the lethal exposure time for 50% mortality range from 117% to 119% when corrected for the given water depths. Comparing results from these studies, Atlantic salmon and brown trout are more susceptible to GBD than Pacific salmonids [30]. However, these results also reveal high intraspecific variations. According to Antcliffe et al. [25], such intraspecific variations suggest that the susceptibility to TDGS differs among stocks and among individual fish within a stock. Additional factors, such as pre-exposure handling stress and genetic variations, might also have contributed to the observed intraspecific differences in tolerance to TDGS. Intraspecific differences in mortality rate to Atlantic salmon parr from trial 1 and trial 3 (reference group) might be a result of differences in flow rate, O_2_:N_2_ ratio, fish size, genetic differences, or a combination of these factors.

**Table 9:** Inter- and intraspecific difference in tolerance of salmonids to total dissolved gas supersaturation.

Specie	L (cm)	T (°C)	D (cm)	TDG	TDG_corr_	LT_50_	References
*S. salar*	16	6	30	120	117	4	Trial 1
*S. trutta*	13	8	30	121	118	12	Trial 3
*O. tshawytscha*	12‡	15	25	121	118	14	[28]
*S. salar*	14	8	30	121	118	15	Trial 3
*S. trutta*	17	10	22	121	118	24	[28]
*S. trutta*	17	11	22	121	118	24	[29]
*O. mykiss*	14	8	30	121	118	25	Trial 3
*O. mykiss*	18	10	28	120	117	30	[35]
*O. mykiss*	18	10	22	121	118	36	[28]
*O. nerka*	10†	10	65	125	118	38	[36]
*O. mykiss*	11†	10	25	122	119	43	[25]
*O. mykiss*	18	10	22	121	118	48	[29]
*O. tshawytscha*	13†	12	28	120	117	60	[27]
*O. kisutch*	10	14	14	119	117	62	[37]
*O. mykiss*	19	10	22	121	118	91	[28]
*O. kisutch*	10‡	14	14	119	117	101	[37]

Tolerance of salmonid parr (*Salmo* spp. and *Oncorhynchus* spp.) to total dissolved gas supersaturation measured as lethal exposure time in hours for 50% mortality (LT_50_). Total dissolved gas (TDG) and TDG corrected for the given compensation depth are expressed as percentages (%). Data as mean fish length (L), water temperature (T), water depth (D), and TDG were derived from the literature (see references) and compared with results from trial 1 and trial 3 in the present study. Fish lengths measured as total length, fork length† or not given‡. The list has been ranked from lowest to highest LT_50_.

### Abiotic factors

Flow rate in trial 1 was set to 2200 L^-hour^ (36.7 L^-min^) in fish tanks holding 300 litres of water, which means that the tank water was replaced 7.3 times per hour. This turnover rate was 5.5 times faster in trial 1 compared to trial 3 that had a flow rate of 400 L^-hour^ (6.7 L^-min^) in tanks with the same water volume. Previous studies suggest that an increased flow rate may cause more severe GBD. For example, exercise or forced swimming activity may increase the risk and severity of GBD development in fish exposed to DGS [34]. Also, the mortality towards TDG in rainbow trout fry (average length 4.5 cm) exposed to 114% TDG increased significantly when the flow rate was changed from 200 mL^-min^ to 400 mL^-min^ [6]. It is therefore reasonable that the higher flow rate in trial 1 with Atlantic salmon parr may have contributed to the higher mortality rate and more severe signs of GBD compared to the repeated trial 3 with Atlantic salmon parr in treatment groups with a lower water flow rate. However, under natural conditions in a river, juvenile Atlantic salmon and brown trout seek shelter between rocks and in vegetation to avoid predators and to reduce energy expenditure [38] and will therefore be less exposed to high flow rates that force high and prolonged swimming activity. The assumption that forced swimming activity may initiate more severe GBD has also been challenged by Pleizier et al. [39] in trials with swimming treatment of rainbow trout exposed to DGS. These swimming treatments showed no statistically significant effect on time to loss of equilibrium in treatments of juvenile rainbow trout exposed to TDG 123% in water with a flow rate of 10.6 L^-min^ (water velocity measured to 23 cm^-s^) compared to static water. Therefore, it was concluded that the flow rate did not have a significant effect on the progression of GBD in rainbow trout.

The O_2_:N_2_ ratio in group T120 (1.08, see Table 1), subjected to TDG pressure of 120% in trial 1, was higher than the O_2_:N_2_ ratio for the same group in trial 3 (0.96). Previous studies have shown that an increased O_2_:N_2_ ratio at 119–120% TDG significantly increases the survival of juvenile coho (mean fish length 6 cm) and sockeye (mean fish length 8.6 cm) when the O_2_:N_2_ ratio exceeds 1.5 [40,37]. Interestingly, results from the experiments conducted by Nebeker et al. [40] also showed that more extensive and severe signs of GBD (subcutaneous emphysema and lesions) were developed at high O_2_:N_2_ ratios. Hence, the relatively high prevalence of fish with bubble formation in fins and the higher mortality rate in Atlantic salmon parr from trial 1 compared to salmonid parr from trial 3 (brown trout, Atlantic salmon, and rainbow trout) may partly be explained by higher O_2_:N_2_ ratio in trial 1 for the group T120 (120% TDG).

### Fish size and life stages

Atlantic salmon parr in trial 1 were significantly larger than the Atlantic salmon parr used in trial 3, and these differences in fish size might have contributed to a higher mortality rate in trial 1. Larger fish exhibit lower tolerance to TDG supersaturation and higher mortality rates than smaller fish [25,29,33,35,37,41–43], but this is most prominent when fish from different life stages are compared [3,26]. Krise & Herman [26] showed that LL50 (lethal level causing 50% mortality) for Atlantic salmon parr (14.8 cm) was mean TDG_corrected_ of 116% (TDG corrected for water depth used in the trial; see Eq. 4), while LL50 for small fish (6–7 cm) ranged from TDG_corrected_ 119% to 122%. In a similar trial, Connor [3] found that brown trout parr exposed to TDG_corrected_ 114.5% reached LT_50_ (lethal time causing 50% mortality) at 24 hours and 48 hours for fish sizes of 17.3 cm and 12.6 cm respectively, but the same trial with rainbow trout parr revealed no significant difference in mortality rate between small (13.1 cm) and larger fish (17.9 cm). These results suggest an interspecific variance in size-dependent tolerance to TDGS for brown trout and rainbow trout. However, both brown trout fry and rainbow trout fry showed significantly higher tolerance to TDGS than parr in this study, which aligns with the findings of Krise & Herman [26]. These findings are also consistent with the results from our trials with Atlantic salmon from different development stages (parr, fry, and alevins). Specifically, mortality rates among Atlantic salmon fry were low (16%) compared to Atlantic salmon parr (>50%) when exposed to TDG_corrected_ 116% for days and hours, respectively. No mortality associated with GBD was observed among the alevins. Furthermore, clinical signs of GBD in fry appeared only after days (subacute) of exposure to TDG_corrected_ 116% in contrast to Atlantic salmon parr where such signs appeared within a few hours (acute) when exposed to TDG_corrected_ ≥ 112%.

Transformation from cutaneous to branchial oxygen uptake occurs during the larval stage. According to Wells & Pinder [44], cutaneous respiration in Atlantic salmon larvae declines from 92% at hatch to 32% by the end of yolk absorption, as cutaneous respiration decreases concomitant with development of gills and an increase of branchial surface. This study [44] showed that the respiratory cutaneous and branchial surface areas are the same for Atlantic salmon at fish sizes between 5 and 6 g wet body, which correspond to fish length 8–9 cm given a Fulton’s K of 1. Development of external gill structures also coincides with the development of other parts of the respiratory- and circulatory system, and there is a coordination when it comes to the development of pumping systems for water and blood through the gills [45]. Development of gills and cardiovascular system continues during the stage of fry (swim-up, first-feeding) as increased activity requires efficient and increased oxygen uptake. Therefore, larger fish with fully developed branchial respiratory systems will be more susceptible to GBD due to more effective gas exchange through gills and gas transport in the vascular system. Overall, our results are consistent with these previous studies showing intraspecific differences in tolerance of TDG supersaturation for different development stages of salmonids. That is, early stages of salmonids (alevin and fry) have higher tolerance to TDG supersaturation than the larger salmonid parr [25,29,37,41].

### Sublethal effects

Our results show that long-term exposure of sublethal levels of TDGS may cause subacute or chronic GBD with similar clinical signs as for acute GBD among salmonid parr, which include subcutaneous emphysema in fins and lateral line, gas emboli in gills, and even haemorrhagic ascites. Chronic GBD (long-term exposure to TDGS) has also been associated with signs, such as fin erosion, lesions or secondary infections, petechial haemorrhages, and ectoparasitic infections among non-salmonid fish [43]. Additionally, long-term exposure of TDGS can lead to exophthalmia and cataract formation [1,3,28,29,35]. It should be noted that these latter signs may persist for several days post-recovery at normal TDG [3, 29, 43, present study] and can therefore be mistaken for eye disorders related to non-infectious causes, infectious diseases, and nutritional eye diseases [45–49].

The response of two juvenile Pacific salmon species (*Oncorhynchus tshawytscha* and *O. mykiss*) to GBD also includes abnormal behaviour, impaired swimming ability, and reduced growth [1,6,8,28,50,51]. In our study, abnormal swimming behaviour, such as erratic swimming patterns and apathy, were most often observed among salmonids and minnows exposed to the highest levels of TDG supersaturation (≥ 115%). Apathetic behaviour, characterized by a lack of flight response to external stimuli, was particularly noted in Atlantic salmon parr (trial 1) that had been exposed to TDG ranging from 107% to 110% for more than a week. Atlantic salmon fry (trial 2) exhibited erratic swimming near the surface, likely caused by gas bubble formation in the gastrointestinal tract leading to positive buoyancy. Similar bubble formation in the gastrointestinal tract has been described in experimental trials with Atlantic salmon fry [5]. In natural settings, behaviours including reduced flight response or increased buoyancy would likely increase the risk of predation. The impact of TDG supersaturation on swimming performance and behaviour in both salmonid and non-salmonid species has been documented in previous studies. Findings suggest that certain species may exhibit reduced swimming capacity not only during exposure but also persisting for days after return to normal TDG conditions [28,52,53].

Alevins from the control group (T100) were significantly larger compared to alevins exposed to TDG ≥ 115% at the end of trial 2. Similarly, brown trout parr (trial 3) exposed to 110% for 21 days (including 7 days recovery) were significantly lighter in weight and showed a lower Fulton’s K compared to the control group (T100). This difference might be attributed to reduced appetite or increased metabolic rate or both, caused by chronic stress. These findings suggest that reduced growth may be an effect of prolonged exposure to sublethal levels of DGS, which is consistent with the findings of Dawley and Ebel [28]. Juvenile stages of salmonids (fry, parr, and smolt) are particularly sensitive to environmental stressors due to their elevated growth rates and high metabolic demands. Chronic or repeated exposure to sublethal stress, such as TDGS, can impair aerobic performance, reduce growth efficiency, and delay developmental transitions including smoltification [54,55]. Reduced physiological condition during these critical periods is strongly associated with lower downstream survival, as stressed smolts exhibit impaired osmoregulatory capacity and diminished predator-avoidance behaviours [10,56–58].

Bubble formation in the lateral line has been described as the most common sign of GBD in salmonids [8,59]. The lateral line sensory system allows fish to detect hydrodynamic stimuli, providing the basis for rapid behavioural responses [60]. Extensive gas bubble formation associated with acute gas bubble disease has been proposed to disrupt this sensory function, reducing responsiveness to stimuli and compromising predator avoidance [10,61]. However, sporadic bubbles in the lateral line may also occur on fish under normal TDG levels [35], aligning with our results. These findings suggest that scattered bubbles covering less than 15% of the lateral line may not be a reliable clinical indicator of GBD. In contrast, bubbles covering more than 15% of the lateral line were easily detected using a dissection microscope in brown trout and rainbow trout parr exposed to DGS in our study. No such bubble formation was detected in the lateral line on Atlantic salmon (trial 1 and trial 3) that were exposed to TDGS ranging from 100% to 120%. Bubble formations in the lateral line on Atlantic salmon exposed to TDG supersaturation are rarely documented in the literature. Nevertheless, such bubble formation might occur when juvenile Atlantic salmon are exposed to TDGS higher than 121% [26].

### Ecological effects

Differences in habitat preference and physiology can explain the variation in tolerance to TDGS among fish species and among life stages. Habitat preferences, such as whether a species typically inhabits lotic (river) or lentic (lake) ecosystems, or favours deep versus shallow zones, can influence both their exposure to and resilience against environmental stressors like TDGS [1,22,34,62–64]. In addition, physiological traits, including variations in gill surface area, haemoglobin oxygen affinity, and metabolic rate, also play a significant role in determining how different species or life stages respond to such challenges [8,44,65].

Interspecific differences in the vulnerability to TDGS have been documented in several studies and meta-analyses [8,30,66,67]. Pleizier et al. [30] highlighted variability in the physiological response to TDGS, noting that cyprinids and other non-salmonid species tend to be more resilient to TDGS and less prone to developing GBD compared to salmonids. The mechanisms for this are unclear, but it may be associated with varying metabolic rates that determine the rate of respiration and incorporation of gases into the bloodstream. In contrast, Atlantic salmon, brown trout, and rainbow trout parr exhibited similar responses to high concentrations of dissolved gases, often displaying physical signs of gas bubble disease.

In the present study, the European minnow (Cypriniformes: Leuciscidae) was selected as a representative non-salmonid species due to its co-occurrence with brown trout (*Salmo trutta*) and Atlantic salmon (*Salmo salar*) in many Norwegian streams and lakes. The European minnow has spread in Norway [68] and is classified as a regionally invasive species with a very high ecological risk [69]. The abundance of native brown trout is on average 35% lower in lakes with minnow [68] due to competition for common food resources and reduced recruitment and growth of brown trout [70,71]. Our trials suggest that adult minnow possess a higher tolerance to TDGS than Atlantic salmonid parr. Overall, these results suggest that in rivers affected by TDGS, minnow are likely to be favoured over salmonids, potentially leading to adverse ecological impacts [64]. This study presents the first assessment of TDGS tolerance in European minnow (*Phoxinus phoxinus*), demonstrating relatively low mortality (16%) following continuous exposure to 120% TDG for 14 days. Current knowledge of TDG sensitivity among Leuciscidae species is limited. Nebeker et al. [72] reported a lethal time (LT₅₀) ranging from 380 to 580 hours for Speckled dace (*Rhinichthys osculus*) at 125% TDGS. In contrast, Beeman et al. [12] documented a substantially shorter LT₅₀ of 15.3 hours for Northern pikeminnow (*Ptychocheilus oregonensis*) and 116 hours for Redside shiner (*Richardsonius balteatus*) under similar TDGS conditions.

Laboratory trials provide the experimental control with which to document the responses of animals to environmental pollutants, however, there are limitations, and such holding studies may represent a worst-case scenario for exposure. TDGS at the water surface declines at deeper depths in the water column, where the hydrostatic pressure will compensate for the surface saturation with a rate of 9.7% TDG per meter in freshwater at sea level [73]. In laboratory trials, tank depth has a significant influence on the exposure of fish to supersaturation and the progress of gas bubble disease. In the field, fish can modulate their exposure to supersaturated water by seeking refuge, moving down- or upriver, across streams, or descending to greater depth. Microturbulence in the water can create refuges that are difficult to detect with static environmental loggers. Field studies of gas supersaturation have not identified consistent responses of animals; Lennox et al. [22] found that brown trout did not exhibit consistent diving responses to manage exposure to gas supersaturation, which is consistent with other surveys of mountain whitefish (*Prosopium williamsoni*) and rainbow trout [74,75].

Group dynamics in tanks may play a role in which specimens experience gas bubble disease and which do not. For species in competition with each other for space, dominant individuals are likely to occupy preferred positions and subordinates may be more prone to the effects of gas supersaturation by not being able to maintain territories in deeper refuge areas. For schooling species, such as minnow, this may help the fish to limit their average exposure across all individuals. We separated the species physically in our study, but combining different species could yield varied outcomes due to interspecific competition for space. Additionally, providing enrichment in the tanks could alter results by offering refuges that mitigate exposure effects.

### Implication for management

Total dissolved gas treatment groups were chosen to emulate common conditions encountered by native fish in boreal rivers where gas supersaturation occurs because of hydropower regulation. The highest concentration of dissolved gases in the groups was 120%, which is well below the level that can occur in regulated rivers. Monitoring suggests that seasonal saturopeaking [19] can exceed 200% DGS, a level that is difficult to replicate in laboratory settings, but likely to cause a higher prevalence and faster onset of GBD. Even low levels of sustained gas supersaturation can lead to ecological disturbance, highlighting the need for stringent monitoring and mitigation measures [63,64,76]. According to Lutz [46], GBD associated fish kills occur in hydropower regulated river system when a) TDG supersaturation exceed 120% over several weeks, b) uncompensated TDG, measured as ∆P, becomes positive (> 100% TDG) at maximum river depth, or c) there is a rapid reduction in discharge from a hydropower plant after saturopeaking. Somewhat surprising, Höglund et al [77] found no indication of stress-on-stress effects between TDGS and acidification, implying that the effects we observed does not need to be accommodated in the presence of acidification, which is common in many parts of Norway and elsewhere.

In our study, experimental trials with Atlantic salmon (trial 1) and brown trout parr (trial 3) have shown that bubble formation in fins or lateral line (only on brown trout) may occur at DGS as low as 105% (or 102% when corrected for compensation depth; TDG_corrected_). Furthermore, our results indicate that prolonged exposure to TDG > 108% (>106% TDG_corrected_) can lead to mortality in parr of Atlantic salmon and brown trout. Consequently, we suggest that TDG in hydropower regulated rivers should not exceed 102% or 106% TDG_corrected_ at any given compensation depth to eliminate or significantly reduce the risk of GBD or mortality among juvenile salmonids, respectively. These thresholds align with Krise & Herman [78], who advocated water TDG below 105% to prevent GBD. Notably, the threshold for Atlantic salmon and brown trout to develop severe clinical signs of GBD symptoms appears lower than compared to rainbow trout and other *Oncorhynchus* species. Fidler and Miller [15] reported that a TDG pressure higher than 115% at sea level is required to initiate bubble growth in the cardiovascular system or gill filaments in juvenile rainbow trout, sockeye, cutthroat trout, and chinook salmon. It is also essential to consider that low-level chronic TDG exposure can stress fish, cause reduced growth [28, present study], reduced swimming performance [11,15,29], and potentially increasing susceptibility to secondary infections causing bacterial or fungal diseases and fin rot [29,79–84]. Furthermore, fish that survive an initial TDGS may become more susceptible to subsequent exposures with increased severity of symptoms upon repeated exposure to TDGS [3,29,81]. This increased susceptibility may be due to the presence of intravascular micronuclei (small gas bubbles) that persist following previous exposures [85]. Results from our study and previous studies have shown that recovery from sublethal levels of TDGS is possible to some extent [1,35]. Subcutaneous emphysema in skin and fins usually resolves or disappear within days or weeks at normal TDG, while recovery from other GBD symptoms, such as exophthalmia, requires a more prolonged period than subcutaneous emphysema [40, present study].

Residual scar tissue in skin and fins suggest permanent tissue damage [3, 29, present study]. White et al. [29] note that such tissue damage weakens the fish, and that repeated exposures will reduce the ability to recover from GBD. Scar tissue formed during recovery (at normal TDG level) between TDGS exposures could accelerate formation of subcutaneous emphysema by acting as nucleation sites [3]. Therefore, fish recovered from GBD will experience a more rapid development of GBD if re-exposed to DGS. Furthermore, Weitkamp [85,86] found that juvenile chinook salmon that died after recovery from GBD had developed fungal infections in their fins, suggesting an increased risk for secondary infections in affected areas post-recovery.

### Conclusion

The study demonstrates that fish tolerance to TDGS varies significantly among species and among life stages in fish. Salmonid parr, especially Atlantic salmon (*S. salar*) and brown trout (*S. trutta*), are particularly vulnerable to acute GBD at TDG levels ≥110%, while earlier life stages, like fry and alevins as well as the European minnow, show much higher tolerance to approx. 120%. Factors, such as water depth, exposure time, flow rate, O₂:N₂ ratio, fish size, and developmental stage likely influence susceptibility to GBD, with larger fish and higher flow rates generally leading to increased mortality. Sublethal TDGS exposure can lead to chronic GBD, reduced growth, abnormal behaviour, and heightened susceptibility to disease and predation. Interspecific comparisons suggest that species, like European minnow, which are more resilient to TDGS, may outcompete sensitive salmonids in affected rivers, potentially altering local ecosystems.

Effective monitoring and mitigation strategies are essential to protect native fish populations in hydropower-influenced environments. Based on the results of this study, it is recommended that TDGS in regulated rivers should not exceed 108% in shallow water (< 0.3 m) or 106% when depth-corrected to prevent GBD and mortality in salmonid parr. To avoid all adverse effects of elevated TDG supersaturation, including sublethal effects, even lower TDG threshold values should be considered, particularly in shallow areas where prolonged exposure over several days or weeks may occur.

## References

[1] Bouck GR. Etiology of gas bubble disease. Trans Am Fish Soc. 1980;109(6):703–7. doi: 10.1577/1548-8659(1980)109<703:eogbd>2.0.co;2

[2] Colt J. Gas supersaturation — Impact on the design and operation of aquatic systems. Aquacult Eng. 1986;5(1):49–85. doi: 10.1016/0144-8609(86)90005-1

[3] Connor WP. Effects of gas supersaturated water on juvenile brown and rainbow trout [M.Sc. thesis]. Montana State University; 1988. Available from: https://scholarworks.montana.edu/handle/1/6569

[4] Fidler LE. Gas bubble trauma in fish [PhD thesis]. University of British Columbia; 1988. doi: 10.14288/1.0097966

[5] Geist DR, Linley TJ, Cullinan V, Deng Z. The effects of total dissolved gas on chum salmon fry survival, growth, gas bubble disease, and seawater tolerance. N Am J Fish Manag. 2013;33(1):200–15. doi: 10.1080/02755947.2012.750634

[6] Machado JP, Garling DL Jr, Kevern NR, Trapp AL, Bell TG. Histopathoiogy and the Pathogenesis of Embolism (Gas Bubble Disease) in Rainbow Trout (*Salmo gairdneri*). Can J Fish Aquat Sci. 1987;44(11):1985–94. doi: 10.1139/f87-243

[7] Rossland BO. Vannkvalitetens betydning for fiskehelsen. In: Poppe T, editor. Fiskehelse og fiskesykdommer. Oslo: Universitetsforlaget AS; 1999. p. 240–52.

[8] Weitkamp DE, Katz M. A review of dissolved gas supersaturation literature. Trans Am Fish Soc. 1980;109(6):659–702. doi: 10.1577/1548-8659(1980)109<659:arodgs>2.0.co;2

[9] Birtwell I, Korstrom J, Komatsu M, Fink B, Richmond L, Fink R. The susceptibility of juvenile chum salmon (*Oncorhynchus keta*) to predation following sublethal exposure to elevated temperature and dissolved gas supersaturation in seawater. Can Tech Rep Fish Aquat Sci. 2001;2343. Available from: https://publications.gc.ca/pub?id=9.579284&sl=0

[10] Mesa MG, Warren JJ. Predator avoidance ability of juvenile chinook salmon (*Oncorhynchus tshawytsoha*) subjected to sublethal exposures of gas-supersaturated water. Can J Fish Aquat Sci. 1997;54(4):757–64. doi: 10.1139/cjfas-54-4-757

[11] Schiewe MH. Influence of dissolved atmospheric gas on swimming performance of juvenile chinook salmon. Trans Am Fish Soc. 1974;103(4):717–21. doi: 10.1577/1548-8659(1974)103<717:iodago>2.0.co;2

[12] Beeman JW, Venditti DA, Morris RG, Gadomski DM, Adams BJ, VanderKooi SP, et al. Gas bubble disease in resident fish below grand coulee dam. Final Report of Research. Cook, Washington: U.S. Geological Survey, Western Fisheries Research Laboratory; 2003. Available from: https://pubs.usgs.gov/publication/70179865

[13] Crew AV, Keatley BE, Phelps AM. Literature review: Fish mortality risks and international regulations associated with downstream passage through hydroelectric facilities. Can Tech Rep Fish Aquat Sci. 2017. Available from: https://publications.gc.ca/pub?id=9.833743&sl=0

[14] Ebel WJ. Supersaturation of nitrogen in the columbia river and its effect on salmon and steelhead trout. Fish Bull. 1969;68(1). Available from: spo.nmfs.noaa.gov/sites/default/files/pdf-content/1970/681/ebel.pdf

[15] Fidler LE, Miller SB. British Columbia water quality guidelines for the protection of aquatic biota from dissolved Gas supersaturation - Technical report. Cranbrook (BC): Aspen Applied Sciences Ltd; 1997. Available from: http://www2.gov.bc.ca/assets/gov/environment/air-land-water/water/waterquality/water-quality-guidelines/approved-wqgs/totalgas-tech.pdf

[16] Li P, Zhu DZ, Li R, Wang Y, Crossman JA, Kuhn WL. Production of total dissolved gas supersaturation at hydropower facilities and its transport: a review. Water Res. 2022;223:119012. doi: 10.1016/j.watres.2022.119012 36041368

[17] Orlins JJ, Gulliver JS. Dissolved gas supersaturation downstream of a spillway II: Computational model. J Hydraul Res. 2000;38(2):151–9. doi: 10.1080/00221680009498350

[18] Pulg U, Vollset KW, Velle G, Stranzl S. First observations of saturopeaking: Characteristics and implications. Sci Total Environ. 2016;573:1615–21. doi: 10.1016/j.scitotenv.2016.09.143 27707575

[19] Pulg U, Lennox RJ, Enqvist M, Stranzl SF, Espedal EO, Schwarz M, et al. Assessing the potential for gas supersaturation downstream of hydropower plants in Norway, Austria and Germany. Sci Total Environ. 2024;948:174645. doi: 10.1016/j.scitotenv.2024.174645 38986702

[20] Qu L, Li R, Li J, Li K, Deng Y. Field observation of total dissolved gas supersaturation of high-dams. Sci China Technol Sci. 2010;54(1):156–62. doi: 10.1007/s11431-010-4217-8

[21] Velle G, Isaksen TE, Lennox RJ, Pulg U. Bubbling trouble: effects of supersaturated water on benthic macroinvertebrates. Ecohydrology. 2024;17(6):e2665. doi: 10.1002/eco.2665

[22] Lennox RJ, Thiemer K, Vollset KW, Pulg U, Stranzl S, Nilsen CI, et al. Behavioural response of brown trout (*Salmo trutta*) to total dissolved gas supersaturation in a regulated river. Ecohydrology. 2021;15(1):e2363. doi: 10.1002/eco.2363

[23] Pulg U, Isaksen TE, Velle G, Stranzl SF, Espedal EO, Vollset KW, et al. Gassovermetning i vassdrag - en kunnskapsoppsummering. Norwegian Research Centre: NORCE LFI; 2018. Rapport nr. 312. Finansiert av Miljødirektoratet (ref. nr. M-1126). Norwegian. Available from: hdl.handle.net/11250/2626856

[24] Stenberg SK, Velle G, Pulg U, Skoglund H. Acute effects of gas supersaturation on Atlantic salmon smolt in two Norwegian rivers. Hydrobiologia. 2020;849(2):527–38. doi: 10.1007/s10750-020-04439-z

[25] Antcliffe BL, Fidler LE, Birtwell IK. Effect of dissolved gas supersaturation on the survival and condition of juvenile rainbow trout (*Oncorhynchus mykiss*) under static and dynamic exposure scenarios. Can Tech Rep Fish Aquat Sci; 2002. Available from: https://publications.gc.ca/pub?id=9.579311&sl=0

[26] Krise WF, Herman RL. Resistance of underyearling and yearling atlantic salmon and lake trout to supersaturation with air. J Aquat Anim Health. 1991;3(4):248–53. doi: 10.1577/1548-8667(1991)003<0248:rouaya>2.3.co;2

[27] Mesa MG, Weiland LK, Maule AG. Progression and severity of gas bubble trauma in juvenile salmonids. Trans Am Fish Soc. 2000;129(1):174–85. doi: 10.1577/1548-8659(2000)129<0174:pasogb>2.0.co;2

[28] Dawley E, Ebel W. Effects of various concentrations of dissolved atmosferic gas on juvenile Chinook salmon and steelhead trout. Fish Bull. 1975;73(4):787–96. Available from: https://spo.nmfs.noaa.gov/sites/default/files/pdf-content/1975/734/dawley.pdf

[29] White R, Phillips G, Liknes G, Brammer J, Connor W, Fidler L, et al. Effects of supersaturation of dissolved gases on the fishery of the Bighorn River downstream of the Yellowtail Afterbay Dam. Completion report. U.S. Fish and Wildlife Service, Montana Cooperative Fishery Research Unit, Montana State University; 1991. Available from: https://biodiversitylibrary.org/page/56388639

[30] Pleizier NK, Algera D, Cooke SJ, Brauner CJ. A meta‐analysis of gas bubble trauma in fish. Fish Fish. 2020;21(6):1175–94. doi: 10.1111/faf.12496

[31] White RG, Philips G, Liknes G, Sprague C, Brammer J, Connor W, et al. The Effects of supersaturation of dissolved gases on the fishery of the Bighorn River downstream of the Yellowtail Afterbay Dam. 1986 annual report. Montana Cooperative Fishery Research Unit, Montana State University; 1987. Available from: https://biodiversitylibrary.org/page/47046341

[32] Baird RB, Rice EW, Eaton AD, Bridgewater L, editors. Standard methods for the eximination of water and wastewater. 23rd ed. Washington (DC): American Public Health Association; 2017. doi: book/10.2105/SMWW.2882

[33] Dawley EM, Monk B, Schiewe M, Ossiander F, Ebel W. Salmonid bioassay of supersaturated dissolved air in water. US Environmental Protection Agency. Ecological Research Series. EPA-600/ 3-76-056; 1976. Available from: www.nepis.epa.gov/Exe/ZyPURL.cgi?Dockey=910170BK.txt

[34] Stroud RK, Nebeker AV, editors. A study of the pathogenesis of gas bubble disease in steelhead trout (*Salmo Gairdneri*). In: Fickeisen DH, Schneider MJ, editors. Gas bubble disease. Report CONF-741033; 1976. p. 66–71. Available from: www.arlis.org/docs/vol1/Susitna/4/APA435.pdf

[35] Dawley E, Schiewe M, Monk B. Effects of long term exposure to Supersaturation of dissolved atmospheric gases on juvenile chinook salmon and steelhead trout in deep and shallow test tanks. In: Fickeisen DH, Schneider MJ, editors. Gas bubble disease. Report CONF-741033; 1976. Available from: www.arlis.org/docs/vol1/Susitna/4/APA435.pdf

[36] Bouck GR, Nebeker AV, Stevens DG. Mortality, saltwater adaption and reproduction of fish during gas supersaturation. Ecological Research Series. Report no. EPA-600/ 3-76-050; 1976. Available from: purl.fdlp.gov/GPO/gpo85199

[37] Rucker RR. Gas bubble disease: mortalities of coho salmon, *Oncorhynchus kisutch*, in water with constant total gas pressure and different oxygen-nitrogen ratios. Fish Bull. 1975;73(4):915–8. Available from: pubs.usgs.gov/publication/70181898

[38] Velle G, Skoglund H, Barlaup BT. Effects of nuisance submerged vegetation on the fauna in Norwegian rivers. Hydrobiologia. 2021;849(2):539–56. doi: 10.1007/s10750-020-04465-x

[39] Pleizier NK, Cooke SJ, Brauner CJ. Does swimming activity influence gas bubble trauma in fish?. River Res Appl. 2022;39(1):65–72. doi: 10.1002/rra.4069

[40] Nebeker AV, Bouck GR, Stevens DG. Carbon dioxide and oxygen-nitrogen ratios as factors affecting salmon survival in air-supersaturated water. Trans Am Fish Soc. 1976;105(3):425–9. doi: 10.1577/1548-8659(1976)105<425:cdaora>2.0.co;2

[41] Jensen JOT, Schnute J, Alderdice DF. Assessing juvenile salmonid response to gas supersaturation using a general multivariate dose–response model. Can J Fish Aquat Sci. 1986;43(9):1694–709. doi: 10.1139/f86-213

[42] Nebeker AV, Andros JD, McCrady JK, Stevens DG. Survival of steelhead trout (*Salmo gairdneri*) eggs, embryos, and fry in air-supersaturated water. J Fish Res Bd Can. 1978;35(2):261–4. doi: 10.1139/f78-043

[43] Saltveit SJ, Brabrand Å, Juárez A, Stickler M, Dønnum BO. The impact of hydropeaking on juvenile brown Trout (*Salmo trutta*) in a Norwegian regulated river. Sustainability. 2020;12(20):8670. doi: 10.3390/su12208670

[44] Wells P, Pinder A. The respiratory development of Atlantic salmon. I. Morphometry of gills, yolk sac and body surface. J Exp Biol. 1996;199(Pt 12):2725–36. doi: 10.1242/jeb.199.12.2725 9320637

[45] Reite OB, Staurnes M, Sigholdt T. Gjellene. In: Døving K, Reimers E, editors. Fiskens fysiologi. Bergen, Norway: John Grieg Forlag AS; 1992. p. 167–73.

[46] Lutz D. Gas supersaturation and gas bubble trauma in fish downstream from a moderately-sized reservoir [M.Sc. thesis]. Iowa State University; 1993. Available from: dr.lib.iastate.edu/handle/20.500.12876/72378

[47] Hughes SG. Nutritional eye diseases in salmonids: a review. Prog Fish-Cult. 1985;47(2):81–5. doi: 10.1577/1548-8640(1985)47<81:nedis>2.0.co;2

[48] Noor El-Deen AI, Shalaby SI, Mona SZ, Abd Elzaher MF. Some infectious and non infectious eye affection syndrome in fish. Life Sci J. 2013;10(2):1362–8. 10.7537/marslsj100213.188

[49] Noor El-Deen AIE, Zaki MS. Eye affection syndrome wild and cultured fish. Life Sci J. 2012;9(3):2568–75. 10.7537/marslsj090312.372

[50] Gültepe N, Ateş O, Hisar O, Beydemir S. Carbonic anhydrase activities from the rainbow trout lens correspond to the development of acute gas bubble disease. J Aquat Anim Health. 2011;23(3):134–9. doi: 10.1080/08997659.2011.616848 22216712

[51] Stroud RK, Bouck GR, Nebeker AV. Pathology of acute and chronic exposure of salmonid fishes to supersaturated water. In: Adams WA, Greer G, Desnoyers JE, Atkinson G, Kell GS, Oldham KB, et al., editors. Chemistry and physics of aqueous gas solutions. Princeton: The Electrochemical Society; 1975. p. 435–49.

[52] Liu X, Li N, Feng C, Fu C, Gong Q, Lai J, et al. Lethal effect of total dissolved gas-supersaturated water with suspended sediment on river sturgeon (Acipenser dabryanus). Sci Rep. 2019;9(1):13373. doi: 10.1038/s41598-019-49800-y 31527649 PMC6746722

[53] Wang Y, Li Y, An R, Li K. Effects of total dissolved gas supersaturation on the swimming performance of two endemic fish species in the upper Yangtze River. Sci Rep. 2018;8(1):10063. doi: 10.1038/s41598-018-28360-7 29968818 PMC6030173

[54] Wedemeyer GA, Barton BA, McLeay DJ. Stress and acclimation. In: Screck C, Moyle P, editors. Methods for fish biology. Bethesda (MD): American Fisheries Society; 1990. p. 415–88. doi: 10.47886/9780913235584.ch14

[55] McCormick SD, Shrimpton JM, Carey JB, O’Dea MF, Sloan KE, Moriyama S, et al. Repeated acute stress reduces growth rate of Atlantic salmon parr and alters plasma levels of growth hormone, insulin-like growth factor I and cortisol. Aquaculture. 1998;168(1–4):221–35. doi: 10.1016/s0044-8486(98)00351-2

[56] Barton BA. Stress in fishes: a diversity of responses with particular reference to changes in circulating corticosteroids. Integr Comp Biol. 2002;42(3):517–25. doi: 10.1093/icb/42.3.517 21708747

[57] Budy P, Thiede GP, Bouwes N, Petrosky CE, Schaller H. Evidence linking delayed mortality of snake river salmon to their earlier hydrosystem experience. N Am J Fish Manag. 2002;22(1):35–51. doi: 10.1577/1548-8675(2002)022<0035:eldmos>2.0.co;2

[58] Hostetter NJ, Evans AF, Roby DD, Collis K. Susceptibility of juvenile steelhead to avian predation: the influence of individual fish characteristics and river conditions. Trans Am Fish Soc. 2012;141(6):1586–99. doi: 10.1080/00028487.2012.716011

[59] Schiewe M, Weber D. Effect of gas bubble disease on lateral line function in juvenile steelhead trout. In: Fickeisen DH, Schneider MJ, editors. Gas bubble disease. Report CONF-741033; 1976. Available from: www.arlis.org/docs/vol1/Susitna/4/APA435.pdf

[60] Bleckmann H, Zelick R. Lateral line system of fish. Integr Zool. 2009;4(1):13–25. doi: 10.1111/j.1749-4877.2008.00131.x 21392273

[61] Weber DD, Schiewe MH. Morphology and function of the lateral line of juvenile steelhead trout in relation to gas‐bubble disease*. J Fish Biol. 1976;9(3):217–33. doi: 10.1111/j.1095-8649.1976.tb04675.x

[62] Weitkamp DE, Sullivan RD, Swant T, DosSantos J. Behavior of resident fish relative to total dissolved gas supersaturation in the lower Clark Fork River. Trans Am Fish Soc. 2003;132(5):856–64. doi: 10.1577/t02-025

[63] Heccbercet TG. Effect of supersaturated water on fish in the River Nidelva, southern Norway. J Fish Biol. 1984;24(1):65–74. doi: 10.1111/j.1095-8649.1984.tb04777.x

[64] Aamold DP. The effects of gas supersaturation on the density of brown trout in two Norwegian rivers [Master thesis]. University of Bergen; 2024. Available from: https://hdl.handle.net/11250/3158640

[65] Perry SF, Gilmour KM. Sensing and transfer of respiratory gases at the fish gill. J Exp Zool. 2002;293(3):249–63. doi: 10.1002/jez.10129 12115900

[66] Maynard C. Evaluation of Total Dissolved Gas criteria (TDG) biological effects research - a literature review. Washington State Department of Ecology Water Quality Program; 2008. 90 p. Available from: https://apps.ecology.wa.gov/publications/documents/0810059.pdf

[67] Xue S, Wang Y, Liang R, Li K, Li R. Effects of total dissolved gas supersaturation in fish of different sizes and species. Int J Environ Res Public Health. 2019;16(13):2444. doi: 10.3390/ijerph16132444 31324054 PMC6651686

[68] Museth J, Hesthagen T, Sandlund OT, Thorstad EB, Ugedal O. The history of the minnow *Phoxinus phoxinus* (L.) in Norway: from harmless species to pest. J Fish Biol. 2007;71(sd):184–95. doi: 10.1111/j.1095-8649.2007.01673.x

[69] Forsgren E, Bærum KM, Finstad AG, Gjelland KØ, Hesthagen T, Knutsen H, et al. Actinopterygii: Vurdering av ørekyt *Phoxinus phoxinus* for Fastlands-Norge med havområder. Fremmedartslista 2023. Artsdatabanken. Norwegian. Available from: www.artsdatabanken.no/lister/fremmedartslista/2023/1767

[70] Borgstrøm R, Museth J, Brittain JE. The brown trout (*Salmo trutta*) in the lake, Øvre Heimdalsvatn: long-term changes in population dynamics due to exploitation and the invasive species, European minnow (*Phoxinus phoxinus*). Hydrobiologia. 2010;642(1):81–91. doi: 10.1007/s10750-010-0161-7

[71] Museth J, Borgstrøm R, Brittain JE. Diet overlap between introduced European minnow (*Phoxinus phoxinus*) and young brown trout (*Salmo trutta*) in the lake, Øvre Heimdalsvatn: a result of abundant resources or forced niche overlap? In: Brittain JE, Borgstrøm R, editors. The subalpine lake ecosystem, Øvre Heimdalsvatn, and its catchment: local and global changes over the last 50 years. New York: Springer; 2010. p. 93–100. doi: 10.1007/s10750-010-0162-6

[72] Nebeker AV, Hauck AK, Baker FD, Weitz SL. Comparative responses of speckled dace and cutthroat trout to air-supersaturated water. Trans Am Fish Soc. 1980;109(6):760–4. doi: 10.1577/1548-8659(1980)109<760:crosda>2.0.co;2

[73] Pleizier NK, Nelson C, Cooke SJ, Brauner CJ. Understanding gas bubble trauma in an era of hydropower expansion: how do fish compensate at depth? Can J Fish Aquat Sci. 2020;77(3):556–63. doi: 10.1139/cjfas-2019-0243

[74] Pleizier NK, Rost-Komiya B, Cooke SJ, Brauner CJ. The lack of avoidance of total dissolved gas supersaturation in juvenile rainbow trout. Hydrobiologia. 2021;848(20):4837–50. doi: 10.1007/s10750-021-04676-w

[75] Algera DA, Kamal R, Ward TD, Pleizier NK, Brauner CJ, Crossman JA, et al. Exposure risk of fish downstream of a hydropower facility to supersaturated total dissolved gas. Water Resour Res. 2022;58(6). doi: 10.1029/2021wr031887

[76] Agostinho AA, Alves DC, Gomes LC, Dias RM, Petrere Jr M, Pelicice FM. Fish die-off in river and reservoir: a review on anoxia and gas supersaturation. Neotrop ichthyol. 2021;19(3):e210037. doi: 10.1590/1982-0224-2021-0037

[77] Höglund E, Loland LZ, Høgberget R, Skov PV, Velle G. No additional stress of sublethal gas supersaturation in a landlocked population of Atlantic salmon (Salmo salar) exposed to environmental acidification. Sci Rep. 2024;14(1):3482. doi: 10.1038/s41598-024-53637-5 38347069 PMC10861563

[78] Krise WF, Herman RL. Tolerance of lake trout, *Salvelinus namaycush* (Walbaum), sac fry to dissolved gas supersaturation. J Fish Dis. 1989;12(3):269–73. doi: 10.1111/j.1365-2761.1989.tb00312.x

[79] Huchzermeyer KDA. Clinical and pathological observations on Streptococcus sp infection on South African trout farms with gas supersaturated water supplies. Onderstepoort J Vet Res. 2003;70:95–105. hdl.handle.net/2263/17682 12967170

[80] Marking LL. Gas supersaturation in fisheries - Causes, Concerns, and Cures. US Department of the Interior, Fish and Wildlife Service Fish and Wildlife Leaflet 9; 1987. p. 1–10. Available from: apps.dtic.mil/sti/tr/pdf/ADA322709.pdf

[81] McGrath KE, Dawley EM, Geist DR. Total dissolved gas effects on fishes of the lower Columbia River. Final report. Report no. PNNL-15525; 2006. Available from: digital.library.unt.edu/ark:/67531/metadc889000

[82] Weiland LK, Mesa MG, Maule AG. Influence of Infection withRenibacterium salmoninarumon Susceptibility of Juvenile Spring Chinook Salmon to Gas Bubble Trauma. J Aquat Anim Health. 1999;11(2):123–9. doi: 10.1577/1548-8667(1999)011<0123:ioiwrs>2.0.co;2

[83] Lutz DS. Gas supersaturation and gas bubble trauma in fish downstream from a midwestern reservoir. Trans Am Fish Soc. 1995;124(3):423–36. doi: 10.1577/1548-8659(1995)124<0423:gsagbt>2.3.co;2

[84] Toner MA, Dawley EM. Evaluation of the effects of dissolved gas supersaturation on fish and invertebrates downstream from Bonneville Dam, 1993. Report to the Portland (OR): U.S. Army Corps of Engineers, Portland District, Contract E96930036; 1995. Available from: www.webapps.nwfsc.noaa.gov/assets/26/7319_07162012_132559_Toner.and.Dawley.1995.pdf

[85] Fidler LE. A study of biophysical phenomena associated with gas bubble trauma in fish [M.Sc. thesis]. University of British Columbia; 1985. doi: 10.14288/1.0096083

[86] Weitkamp DE. Dissolved gas supersaturation: live cage bioassays at Rock Island Dam, Washington. In: Fickeisen DH, Schneider MJ, editors. Gas bubble disease. Report CONF-741033; 1976. Available from: www.arlis.org/docs/vol1/Susitna/4/APA435.pdf

